# A good start in life is important—perinatal factors dictate early microbiota development and longer term maturation

**DOI:** 10.1093/femsre/fuaa030

**Published:** 2020-08-21

**Authors:** Shaopu Wang, Muireann Egan, C Anthony Ryan, Patrick Boyaval, Eugene M Dempsey, R Paul Ross, Catherine Stanton

**Affiliations:** APC Microbiome Ireland, Cork, Ireland, P12 YT20; Teagasc Food Research Centre, Moorepark, Fermoy, County Cork, Ireland, P61 C996; APC Microbiome Ireland, Cork, Ireland, P12 YT20; Teagasc Food Research Centre, Moorepark, Fermoy, County Cork, Ireland, P61 C996; APC Microbiome Ireland, Cork, Ireland, P12 YT20; Department of Paediatrics & Child Health, University College Cork, Cork, Ireland, T12 YN60; DuPont Nutrition & Biosciences, Danisco France SAS - DuPont, 22, rue Brunel, F- 75017 Paris, France; APC Microbiome Ireland, Cork, Ireland, P12 YT20; Department of Paediatrics & Child Health, University College Cork, Cork, Ireland, T12 YN60; APC Microbiome Ireland, Cork, Ireland, P12 YT20; APC Microbiome Ireland, Cork, Ireland, P12 YT20; Teagasc Food Research Centre, Moorepark, Fermoy, County Cork, Ireland, P61 C996

**Keywords:** prenatal and postnatal factors, gut microbiome, transmission, early life, infant, diseases

## Abstract

Maternal health status is vital for the development of the offspring of humans, including physiological health and psychological functions. The complex and diverse microbial ecosystem residing within humans contributes critically to these intergenerational impacts. Perinatal factors, including maternal nutrition, antibiotic use and maternal stress, alter the maternal gut microbiota during pregnancy, which can be transmitted to the offspring. In addition, gestational age at birth and mode of delivery are indicated frequently to modulate the acquisition and development of gut microbiota in early life. The early-life gut microbiota engages in a range of host biological processes, particularly immunity, cognitive neurodevelopment and metabolism. The perturbed early-life gut microbiota increases the risk for disease in early and later life, highlighting the importance of understanding relationships of perinatal factors with early-life microbial composition and functions. In this review, we present an overview of the crucial perinatal factors and summarise updated knowledge of early-life microbiota, as well as how the perinatal factors shape gut microbiota in short and long terms. We further discuss the clinical consequences of perturbations of early-life gut microbiota and potential therapeutic interventions with probiotics/live biotherapeutics.

## Teaser

Perinatal factors impact the health of offspring through modulating the gut microbiota in mothers and infants, which leaves a lasting impression beyond infancy until childhood and adulthood.

## INTRODUCTION

The first years of life are characterised by rapid growth and development of the infant, including physical growth, development of the immune system, and motor, cognitive and behavioural skills. In parallel, the acquisition, selection and colonisation of microbiota residing in the gastrointestinal tract as well as interactions with the host take place until homeostasis is achieved. Increasing evidence points to a mutualistic relationship between early-life gut microbiota and host health in the short and long terms. Although the mechanism and/or causality remain unclear, the accepted dogmatic belief indicates that the disrupted microbiota in early or later life can leave a lasting and potential footprint on health, with a subsequent risk of disease through an altered immune system (Gensollen *et al*. 2016). In comparison to adults, the assembly of the microbiota in early life is susceptible to influences from various factors (Tamburini *et al*. 2016), and a longer length of recovery time from the exposed factors is required (Yassour *et al*. 2016).

During pregnancy, the mother supports foetal development by transferring molecular nutrients and microbial molecules (e.g. short-chain fatty acids and bile acids) to the foetus through the umbilical cord (Macpherson, de Agüero and Ganal-Vonarburg 2017; Ganal-Vonarburg, Hornef and Macpherson 2020). It has been realised that any abnormal changes related to the mother during pregnancy and lactation are linked to the growth and health of the foetus and infant after birth. An important underlying contributor is the gut microbiota of both mother and infant. Numerous factors (collectively named ‘perinatal factors’) have been documented to influence the maternal and infant gut microbiota during pregnancy and lactation, mainly including maternal diet, antibiotic use, mode of delivery, breastfeeding and maternal stress (Table 1). These factors can shape the initial microbiota in infants by altering the composition and diversity of maternal microbiota *per se* that is transmitted to the infant (e.g. maternal gestational diet), or disrupting the vertical mother-to-infant microbial transmission (e.g. mode of delivery), thus predisposing the infants to colonisation by aberrant microbiota, such as opportunistic pathogens (Shao *et al*. 2019). Importantly, these perturbations in the early-life microbiota can persist beyond infancy until childhood and adulthood.

**Table 1.** Summary of selected studies linking perinatal factors dictating early microbiota development and longer term maturation in humans.

Perinatal factors	Cohort characteristics	Time/age at evaluation	Outcomes	Study
Maternal nutrition	81 US mother–infant dyads	Mother: third trimester; infants: 1–2 days and 4–6 weeks	Association of maternal high-fat diet with the gut microbiota of infants at birth and 4–6 weeks of age	Chu *et al*. (2016a)
	145 US mother–infant dyads	Mother: 24–28 gestational weeks; infants: 6 weeks	Association of maternal diet with the gut microbiota of infants at 6 weeks of age dependent on mode of delivery	Lundgren *et al*. (2018)
Antibiotic use	40 Spanish mother–infant dyads [22 no antibiotics, 18 IAP (intrapartum antibiotic prophylaxis)]	Infants: 2, 10, 30 and 90 days	An altered establishment pattern of gut microbiota in IAP infants within the first weeks of life, and a delay in the increase of faecal acetate level in IAP infants	Nogacka *et al*. (2017)
	20 Italian mother–infant dyads (10 no antibiotics, 10 IAP)	Infants: 6–7 days	Infants from IAP group with decreased richness and diversity of gut microbiota, with a lower abundance of Actinobacteria and Bacteroidetes and higher Proteobacteria and Enterobacteriaceae family	Aloisio *et al*. (2016)
	63 Finnish mother–infant dyads (32 no antibiotics, 31 IAP)	Infants: 1 day; 6 months	Influences of IAP on gut microbiota in infants lasting until 6 months after birth	Tapiainen *et al*. (2019)
	16 Finnish mother–infant dyads (8 no antibiotics, 8 IAP)	Infants: 1 and 6 months	IAP affecting the antibiotic resistance genes and mobile genetic element composition until 6 months of life	Pärnänen *et al*. (2018)
	36 Australian mother–infant dyads (13 no antibiotics, 23 intrapartum antibiotic use)	Infants: 3 days	The infant oral microbiota mainly from maternal oral microbiota; and antibiotic treatment at delivery shaping the initial oral microbiome in neonates	Gomez-Arango *et al*. (2017)
Preterm birth	84 US preterm infants	Infants: 6–158 days	Preterm infants harbouring 10% bacterial species of full-term infants; *Enterococcus faecalis*, *Enterobacter cloacae*, *Staphylococcus epidermidis*, *Escherichia coli*, *Klebsiella pneumoniae* and *Klebsiella oxytoca* present in 99.8% samples with high abundance	Gibson *et al*. (2016)
	58 US preterm infants	Infants: 1 day to 11 weeks	Preterm infants mainly harbouring Bacilli, Gammaproteobacteria and Clostridia, and each of the three classes representing the preponderance at different period of life, with increasing proportions of Clostridia at the cost of Bacilli	La Rosa *et al*. (2014)
	39 Irish preterm infants	Infants: 1–36 weeks	Gut microbiota in gut and metabolite in urine of infants changed in a gestational age-dependent manner	Hill *et al*. (2017)
	23 Irish preterm infants	Infants: 1, 2 and 4 years	Impact of gestational age at birth on gut microbiota up to 4 years of age	Fouhy *et al*. (2019)
	45 US preterm infants	Infants: 1–2 days	Preterm-associated bacteria expressing a series of proinflammatory cytokines into vaginal fluid, which was proposed to induce the preterm birth	Fettweis *et al*. (2019)
Caesarean section (C-section)	10 Venezuelan infants (4 vaginally, 6 C-section)	Infants: 1 day	C-section infants harbouring microbiota similar to those on maternal skin surface; vaginally born infants resembling their own mother's vaginal microbiota	Dominguez-Bello *et al*. (2010)
	75 US infants (53 vaginally, 22 C-section)	Infants: 1 day; 4–6 weeks	Mode of delivery clustering microbiota of the oral cavity, nares and skin but not the meconium at birth; no influence at 6 weeks for any body site	Chu *et al*. (2017)
	43 US infants (24 vaginally, 19 C-section)	Infants: 1 day; 1–24 months	C-section-born infants having greater diversity, richness and evenness of gut microbiota in meconium, but declining during the first month and displaying lower diversity and richness up to 2 years of age compared with vaginally born infants	Bokulich *et al*. (2016)
	596 UK infants (314 vaginally, 282 C-section)	Infants: 4, 7 and 21 days; 4–12 months	C-section as the main factor shaping the gut microbiota in early life increasing the opportunistic pathogen colonisation	Shao *et al*. (2019)
	16 Luxembourg infants (7 vaginally, 9 C-section)	Infants: 1, 3 and 5 days	C-section disrupting the mother-to-infant microbial transmission and changing the associated functions	Wampach *et al*. (2018)
Breastfeeding	98 Swedish infants	Infants: <1, 4 and 12 months	Exclusively breastfeeding delaying the maturation of microbiota in infants	Bäckhed *et al*. (2015)
	903 infants (Germany, Sweden, Finland)	Infants: 3–46 months	Breastfeeding dominating the microbial development as measured from 3 to 14 months of life	Stewart *et al*. (2018)
Maternal stress	56 Dutch mother–infant dyads (28 low and 28 high prenatal stress of mothers)	Infants: <110 days	Infants of mothers with high cumulative stress during pregnancy harbouring a higher microbial diversity with enriched Proteobacteria and less lactic acid bacteria and Actinobacteria; the altered microbial colonisation pattern increasing the risk of gastrointestinal symptoms and allergic reactions in infants	Zijlmans *et al*. (2015)

Here, we review the existing findings regarding the influence of perinatal factors on the health of infants to provide a comprehensive overview, which thus far is still lacking. Then, we describe the updated knowledge of the development and maturation of the gut microbiota in early life, including bacteria, viruses and fungi, and pay attention to impacts of perinatal factors on the gut microbiota in early life in short and long terms. Finally, we depict associations between early-life gut microbiota and disease, and discuss the potential therapeutic interventions with probiotics.

## PERINATAL FACTORS DURING PREGNANCY, BIRTH AND POSTPARTUM

During pregnancy, simultaneous changes in hormone secretion, immunity and metabolism in the female take place until delivery and beyond, the process of which interplays with placental development and foetus growth. Perinatal factors mainly include maternal nutrition, antibiotic use, maternal stress and maternal age during pregnancy, as well as gestational age, mode of delivery and breastfeeding, which can interfere with the course of pregnancy and the health outcomes of offspring, in addition to the gut microbiota in early life of the offspring (as discussed below), likely leaving a lasting impression on the risk of disease in later life.

### Maternal nutrition

The macro- and micronutrients during pregnancy provide the essential nutrition for growth of the foetus. Under- and overnutrition as well as diet composition and quality can influence the *in utero* nutrient levels that are associated with the placental and foetal immune development, thereby leading to adverse pregnancy outcomes. For example, in the human, the profile and intake (e.g. proteins, starch and fatty acids) of maternal macronutrients during pregnancy have been shown to be related to foetal body composition and fat distribution (Blumfield *et al*. 2012). Maternal dietary pattern during pregnancy dominated by intake of poultry, nuts, cheese, fruits, whole grains, added sugars and solid fats was associated with greater gestational weight gain but not newborn fat mass or adiposity. The other dietary pattern with an intake of eggs, starchy vegetables and non-whole grains led to a higher maternal fasting glucose and greater newborn adiposity (Starling *et al*. 2017). In addition, the influence of maternal diet during pregnancy on neonatal health could leave a lasting impression on the body composition of offspring (Chen *et al*. 2016; Crume *et al*. 2016). For example, a higher maternal energy intake during pregnancy mainly from carbohydrates and fat led to an increase in neonatal fat mass and risk of adiposity within 3 days after birth (Crume *et al*. 2016). Insufficient micronutrients during pregnancy, for example vitamin D, could reduce the whole-body and lumbar-spine bone-mineral content in children 9 years old (Javaid *et al*. 2006) and might increase the risk of wheeze symptoms in children 12 years old (Devereux *et al*. 2007).

### Antibiotic use

Although prescription of a course of antibiotics to a pregnant woman is a contentious issue, the average number of medications (e.g. antibiotics) that pregnant women took increased from 2.5 in 1976 to 4.2 in 2008 at any period of pregnancy, and >90% of pregnant women took at least one medication in 2008 (Mitchell *et al*. 2011). Although the benefits of antibiotics during and after pregnancy cannot be denied, one concern about antibiotic intervention in pregnant and lactating women is the substances that can be transferred to the foetus via the placenta and umbilical vein by simple diffusion and blood flow. The concentration of antibiotics (e.g. ampicillin, cephalothin and clindamycin) in the umbilical blood reaches a peak within 1 h after maternal serum peak during pregnancy (Chow and Jewesson 1985). Due to the limited activity of foetal hepatic drug-metabolising enzymes compared with adults, the non-metabolised drug thus accumulates in the foetal tissues (Morgan 1997). During lactation, the transfer of maternal antibiotics to the newborn still occurs via breastfeeding as the maternal antibiotics can circulate into breast milk in multiple ways, including simple diffusion, active transport and pinocytosis, depending on the type of antibiotics (e.g. ampicillin, penicillin and cephalosporins) and circulation conditions (Reali *et al*. 2005).

Furthermore, exposure to antibiotics during pregnancy for either mother or foetus or newborn has been associated with an increased risk of multiple diseases, which may be attributed to increased expression of antibiotic resistance genes and altered gut microbiota (Mueller *et al*. 2015; Yassour *et al*. 2016; Neuman *et al*. 2018). For example, at 7 years of age, children whose mothers were exposed to antibiotics during the second or third trimester exhibited 84% higher risk of obesity, compared with children of non-exposed mothers (Mueller *et al*. 2015). Other studies suggest that antibiotic exposure during pregnancy may increase the risk of inflammatory bowel disease among 827 239 Swedish children (Örtqvist *et al*. 2019) and wheeze/asthma in childhood, as suggested by a cohort of 411 Danish children (Stensballe *et al*. 2013) and a meta-analysis (Zhao *et al*. 2015).

### Gestational age

The global incidence of preterm birth (gestational age <37 weeks) is estimated to be up to 15 million each year, which is still rising and accounts for 5–18% of all births across 184 countries, with significant disparities in the frequency of preterm birth among countries [Purisch and Gyamfi-Bannerman 2017; World Health Organization (WHO) 2019]. Two-thirds of preterm births take place after the spontaneous onset of labour (Romero, Dey and Fisher 2014), which is responsible for over 50% of perinatal mortalities and morbidity below the age of 5 years (Goldenberg *et al*. 2008). However, the underlying mechanism for spontaneous preterm birth remains enigmatic. Of note, numerous causes, including infection or inflammation, environmental exposure, immune status, maternal and foetal genome, uteroplacental ischaemia or haemorrhage, maternal anxiety and stress, a decline in progesterone action and changes in microbiota, have all been considered as potential causes with an induction of spontaneous preterm delivery (Goldenberg *et al*. 2008; Romero, Dey and Fisher 2014). Among these determinants, infection and inflammatory responses seem to be the significant risk factors for preterm birth as elevated production of proinflammatory cytokines is associated with uterine activation and preterm birth (Cappelletti *et al*. 2016). Mother's genetics may be a grounded factor that influences the gestation period and the risk of preterm birth (Bezold *et al*. 2013). A genome-wide association study involving a total of 8 643 women found 6 genomic loci that were associated with gestational duration and 3 of these loci were involved with preterm birth. The functions of these genes were involved in uterine development, maternal nutrition and vascular control (Zhang *et al*. 2017). The advances of next-generation sequencing and metagenomic analysis have uncovered the potential association between preterm birth and specific microbiota in pregnancy (as discussed below) (Vinturache *et al*. 2016; Fettweis *et al*. 2019).

### Mode of delivery

Over the years, the occurrence of Caesarean section (C-section) delivery has been unprecedentedly and steadily increasing to 18.6% of all births in 150 countries (Betrán *et al*. 2016) although the threshold of 10–15% was recommended by the WHO in 1985 (WHO 1985). Regionally, the highest proportion of 40.5% was in Latin America and the Caribbean region, followed by 32.3% in North America, 31.1% in Oceania, 25% in Europe, 19.2% in Asia and 7.3% in Africa (Betrán *et al*. 2016). Many efforts, including clinical and non-clinical interventions, have been made in order to reduce unnecessary C-section births, as there is an increasing body of evidence showing the association of short- and long-term risks from C-section with the health of the woman, baby and subsequent pregnancies. Mothers who gave birth via C-section had a decreased risk of urinary incontinence and pelvic organ prolapse, but an increased risk of placenta previa, placenta accrete and placental abruption, as well as miscarriage and stillbirth for subsequent pregnancy (Keag, Norman and Stock 2018). Children delivered by C-section had increased risk of asthma up to the age of 12 years and obesity up to 5 years of age (Keag, Norman and Stock 2018), which was consistent with other studies (Li, Zhou and Liu 2013; Kuhle, Tong and Woolcott 2015; Peters *et al*. 2018). Increased risks of subsequent subfertility, pelvic adhesion and small bowel obstruction have also been observed in mothers who gave birth via C-section (Gurol-Urganci *et al*. 2013; Abenhaim *et al*. 2018; Sandall *et al*. 2018). In addition, C-section could increase risk of overweight and obesity in childhood compared with infants delivered vaginally (Li, Zhou and Liu 2013).

### Breastfeeding

Breast milk contains all the essential macro- [e.g. caseins, whey, fatty acids, human milk oligosaccharides (HMOs) and lactose] and micronutrients (e.g. Immunoglobulin A, Immunoglobulin G, Immunoglobulin M, calcium and vitamin A) (Andreas, Kampmann and Le-Doare 2015; Ahern *et al*. 2019). Based on the nutritional composition, breast milk is commonly classified into colostrum (first milk after birth), transitional milk and mature milk, which also differ in microbiota composition (Gomez-Gallego *et al*. 2016). Colostrum contains high concentrations of whey protein and low concentrations of both lactose and fat compared with mature milk. The nutritional composition of breast milk is dynamic (Andreas, Kampmann and Le-Doare 2015). In general, the content of protein in breast milk gradually decreases with a gradual increase in the concentration of lipid, and lactose production is highest in the fourth to seventh month (Andreas, Kampmann and Le-Doare 2015). The HMOs are an important part of the carbohydrate fraction of human milk, mainly consisting of 2′-fucosyllactose (20–30% of all HMOs) (Ahern *et al*. 2019).

During lactation, exclusive breastfeeding can meet all of the nutritional requirements for the infant's growth. In addition, the bioactive molecules (e.g. HMOs) and microbiota in breast milk help to guide the development and maturation of the infant immune system, promote the colonisation of beneficial microbiota and protect from invasive pathogenic bacteria (Gomez-Gallego *et al*. 2016). In comparison with the infant fed with formula, breastfed infants have lower incidences of various infectious morbidity and diseases, such as necrotising enterocolitis (NEC), respiratory tract infection and decreased risk of childhood obesity and diabetes (Salone, Vann and Dee Stuebe 2009; 2013).

### Other perinatal factors

Chronic psychological distress of mothers (e.g. depression, anxiety and perceived stress) during pregnancy may increase the risk of adverse birth outcomes, including preterm birth (Coussons-Read *et al*. 2012), low birth weight (Lewis, Austin and Galbally 2016) and adverse child neurodevelopment (Sawyer *et al*. 2019). The intergenerational transmission of depression is mainly influenced by genetic inheritance, maternal antidepressant use during pregnancy and childhood maltreatment (Sawyer *et al*. 2019), as well as gut microbiota (Wang *et al*. 2020). In addition, maternal smoking during pregnancy is a potential cause of a range of behavioural problems and disorders in the offspring as the foetus is particularly vulnerable to numerous tobacco components compared with adults (Talati *et al*. 2016).

Due to changes in socioeconomic circumstances and lifestyle in recent years, the proportion of women giving birth after 35 years of age, considered advanced maternal age, has been rising considerably. Both mothers of early age (typically <17 years) at first childbirth and those of advanced age have been realised to be the most vulnerable to infant mortality and poor health outcomes for children and mothers, including high risk of foetal growth restriction, placental abruption, preterm birth, low birth weight, stillbirth, C-section, neonatal intensive care unit (NICU) admission and postnatal morbidity and mortality in children, as well as gestational hypertension and diabetes mellitus for advanced age mothers-to-be (Finlay, Özaltin and Canning 2011; Gibbs *et al*. 2012; Lean *et al*. 2017; Londero *et al*. 2019).

## FEATURES OF THE INFANT GUT MICROBIOTA

### Composition and development of the bacterial population in the infant gut

The infant gut microbiota contains members from the three domains of life, Archaea, Bacteria and Eukarya, as well as viruses. The majority of studies of the infant gut microbiota have utilised 16S ribosomal ribonucleic acid (rRNA) amplicon sequencing to profile the bacterial composition, which are limited by factors such as primer design and sample processing, leading to the underrepresentation of some of the key members of this population (Claesson *et al*. 2010; Hill *et al*. 2016). More recent studies have replaced 16S with whole-genome shotgun sequencing, which is more cost prohibitive but allows for higher taxonomic and functional analyses.

It has been long believed that bacterial colonisation of the infant gut begins at birth but recent studies have questioned if it actually begins *in utero* (as discussed below). Although the infant gut microbiota tends to have lower diversity and as much as six times fewer operational taxonomic units (OTUs) than adults, it is also more dynamic with rapid development over the first 6 months of life (Avershina *et al*. 2016; Yassour *et al*. 2016; Hill *et al*. 2017). The microbiota in infants is mostly represented by four phyla, namely Actinobacteria (genus *Bifidobacterium*), Proteobacteria (genus Enterobacteriaceae—unclassified), Firmicutes (e.g. genera of *Streptococcus* and *Enterococcus*) and Bacteroidetes (genus *Bacteroides*), which normally, with the exception of the Firmicutes, are represented by no more than one genus as indicated in the bracket (Dogra *et al*. 2015).

Immediately after birth, facultative and aerotolerant microorganisms such as Proteobacteria (genus Enterobacteriaceae—unclassified) and Firmicutes (genus *Streptococcus*) dominate, resulting in the depletion of oxygen in the gut and subsequent colonisation by strict anaerobes (Del Chierico *et al*. 2015; Dogra *et al*. 2015). However, studies vary on the exact timing of when such strict anaerobes become dominant. In a recent study, Shao *et al*. (2019) found that by the first 7 days, the microbiota of vaginally born infants was dominated by strict and facultative anaerobes, namely the genera *Bifidobacterium*, *Escherichia*, *Bacteroides* and *Parabacteroides*. Likewise, Hill *et al*. (2017) found that the *Bifidobacterium* genus was dominant 1 week after birth in vaginally born infants, while Jost *et al*. (2012) found that bifidobacteria were significantly more abundant between days 4 and 6. However, other studies have found that facultative aerobes such as Enterobacteriaceae could be dominant for up to 1 or 3 months before being supplanted by *Bifidobacterium* and *Bacteroides* (Dogra *et al*. 2015; Bokulich *et al*. 2016; Yassour *et al*. 2016). However, the majority of studies are in agreement that the Actinobacterium phylum (genus *Bifidobacterium*) becomes dominant between 3 and 6 months of age (Yatsunenko *et al*. 2012; Jakobsson *et al*. 2014; Dogra *et al*. 2015). The early bifidobacterial population at 1 month mainly consists of *Bifidobacterium longum*subsp.*longum*, *Bifidobacterium breve*, *Bifidobacterium bifidum*, *B. longum*subsp.*infantis*, *Bifidobacterium adolescentis* and *Bifidobacterium pseudocatenulatum* species (Duranti *et al*. 2017).

A key point in the development of the infant gut microbiota is weaning and the introduction of solid foods. The introduction of solid foods between 6 and 24 months led to a decrease in *Bifidobacterium* and *Clostridium*, and an increase in *Bacteroides, Faecalibacterium* and Clostridiales revealed by the longitudinal study of 43 US infants from birth to 2 years (Bokulich *et al*. 2016) and 39 Finnish infants from 2 months to 3 years (Yassour *et al*. 2016). On the contrary, a Danish longitudinal study found a significant increase of species within the Bacteroidetes (genus *Bacteroides*) phylum in line with weaning (Bergström *et al*. 2014). This study also noted that while the relative abundance of the *Bifidobacterium* genus decreased with weaning, certain species namely *B. adolescentis* and *Bifidobacterium catenulatum* actually increased in relative abundance (Bergström *et al*. 2014). This is likely due to the decrease of HMO-utilising bifidobacteria (e.g. *B. bifidum*) and an increase in species that are adapted to the utilisation of plant-derived fibres (Egan and van Sinderen 2018). The cessation of breastfeeding leads to an increase in butyrate-producing bacteria, such as *Clostridium leptum*, *Clostridium coccoides* and Lachnospiraceae, possibly associated with energy harvest to new food sources (Bergström *et al*. 2014).

By 2 to 3 years of age, the infant microbiota almost fully resembles the adult-like microbiota (Yatsunenko *et al*. 2012). Alpha diversity increases with age, in particular between 1, 2 and 4 years (Fouhy *et al*. 2019). At 2 years of age, the number of OTUs is almost two-thirds that of adults (Avershina *et al*. 2016), while the composition is also altered, with higher numbers of class Clostridia and *Bacteroides* and a decrease in the relative abundance of bifidobacteria (Bergström *et al*. 2014; Avershina *et al*. 2016). Interestingly, it was noted that the 10 most prevalent OTUs in the infant gut up to 1 year could not be identified in the mother, but at 2 years the most prevalent OTUs were found at the same level in the mother's microbiota (Avershina *et al*. 2016). In another study, two notable bacteria in the adult gut, namely *Akkermansia muciniphila* and *Faecalibacterium prausnitzii*, were detectable at 2 year and 1 year, respectively, having been absent in the first 2 months of life (Yassour *et al*. 2016). However, it has to be mentioned that the absence of microbes may be attributed to the limitation of current approaches, even with shotgun metagenomic sequencing. By 4 years of age, the bacterial profile is dominated by Ruminococcaceae, *Dialister*, *Faecalibacterium*, *Bacteroides* and Christensenellaceae (Fouhy *et al*. 2019).

The concept of enterotypes in the adult gut microbiota was introduced in 2011, when meta-analysis of faecal metagenome studies revealed that individuals could be grouped into three clusters or ‘enterotypes’ based on the relative abundances of *Bacteroides*, *Prevotella* and *Ruminococcus* in their microbiota (Arumugam *et al*. 2011). Bergström *et al*. (2014) studied the *Bacteroides*/*Prevotella* ratio in infants aged between 9 and 36 months and found that such enterotypes were established during this time. Interestingly, other correlations between certain families and genera have also been observed. Notably, there have been incidences of an inverse correlation between the levels of *Bacteroides* and *Bifidobacterium* (Jost *et al*. 2012; Yassour *et al*. 2016), perhaps due to competition for HMOs. Likewise, *B. breve* has been associated with delayed colonisation of certain Clostridia OTUs (Avershina *et al*. 2016). *Bacteroides* have also been negatively correlated with *Clostridium* (Nakayama *et al*. 2011). At a species level, *B. longum* was shown to co-exist with *Lactobacillus* and *Enterococcus* at 9 months, but this had ceased by 18 and 36 months (Bergström *et al*. 2014).

### Sources of gut bacterial population in early life

An important source of gut bacteria for the infant is the mother, in a concept known as vertical transmission (Wang *et al*. 2020). The microbiota can be vertically transmitted from the maternal faecal microbiota, the vaginal microbiota and breast milk. In the faecal samples of vaginally born infants, it was found that over half the bacterial species present in the infant gut on one day of life originated from the mother, with the majority from the mother's gut but also from the vagina, oral cavity and skin in the 25 Italian infants cohort (Ferretti *et al*. 2018), and the mother–infant shared species proportion reached up to 72% in the 98 Swedish infants cohort in the first few days of life (Bäckhed *et al*. 2015).

Species from *Bacteroides*, *Parabacteroides*, *Escherichia* and *Bifidobacterium* were also shown to be vertically transmitted from the maternal faecal microbiota to the infant. Jost *et al*. (2012) have shown that strains of *Bacteroides fragilis* and *Bacteroides stercoris* can be isolated from maternal faecal samples and the corresponding infant stool. Yassour *et al*. (2018) made a particularly interesting observation in that strain diversity within faecal species was higher in the mother than the infant. However, when it came to vertical transmission, it was not always the dominant strain (identified as typically at least 70% relative abundance of that species) that was transferred to the infant. In the case of *Bacteroides dorei*, the secondary strain was more likely to be found in the infant faecal sample. The authors also noted that strains transmitted from the mother were more likely to persist in the infant than strains of the same species from elsewhere.

Bifidobacteria are also commonly vertically transferred from mother to infant, perhaps unsurprisingly given their dominance in the early infant gut. The bifidobacterial species transmitted from mothers’ stool or milk to infants in the first month were identified as *B. bifidum*, *B. adolescentis*, *Bifidobacterium dentium*, *B. breve*, *B. longum* spp. and *B. pseudocatenulatum* (Duranti *et al*. 2017). Likewise, in a separate study, strains of *B. breve* and *B. bifidum* were found in maternal and infant faecal samples, 10 days after birth (Avershina *et al*. 2016).

The vertical transmission of bacteria from breast milk to infants occurs during lactation. In the first month of life, infants who were mainly breastfed received 27.7% of their gut bacterial operational taxonomic units (OTUs) from breast milk (Pannaraj *et al*. 2017), and identical strains of *B. breve* and *Lactobacillus plantarum* were identified in both breast milk and the corresponding infant stool using a culture-dependent method (Murphy *et al*. 2017). Identical strains of *B. bifidum*, *Coprococcus comes* and *Ruminococcus bromii* were identified in breast milk and the corresponding infant stool in an Italian cohort with shotgun metagenomic sequencing (Asnicar *et al*. 2017). Aside from vertical transmission, breast milk can also promote the development of the infant microbiota via HMOs. A number of commensal members of the gut microbiota have been shown to utilise HMOs, including bifidobacteria and *Bacteroides* (Marcobal *et al*. 2011; Egan *et al*. 2014; Duranti *et al*. 2017). The genomes of bifidobacteria (in particular *B. longum* subsp. *infantis*) contain specific genes that are responsible for encoding enzymes that can metabolise HMOs (e.g. fucosidase, *β*-hexosaminidase, sialidase and *β*-galactosidase) and elements for internalisation of HMOs (e.g. permeases for ABC transport system) (Sela *et al*. 2008; Turroni *et al*. 2018). The utilisation of HMOs by *Bacteroides* (e.g. *B. fragilis* and *Bacteroides thetaiotaomicron*) involves the upregulation of mucin glycan degradation pathways (Marcobal *et al*. 2011).

In a separate study, the presence of *Lactobacillus* and *Prevotella* in the newborn's gut was ascribed to these being the predominant genera in the vaginal microbiota. (Dominguez-Bello *et al*. 2010). Similarly, *Lactobacillus* and bifidobacterial OTUs were also found to be shared between the vaginal microbiota and infant faecal samples (Yassour *et al*. 2016).

The hypothesis of *in utero* colonisation and a placental microbiota is relatively recent but also controversial. Bacteria have been found in the placenta, amniotic fluid and the umbilical cord (Wang *et al*. 2013; Aagaard *et al*. 2014; Collado *et al*. 2016). One study identified several genera that were present in the placenta and amniotic fluid as well as in the meconium (the first stool from newborn after birth) of the infant, indicating vertical transmission between placenta and infant (Collado *et al*. 2016). However, the validity of such research has been questioned, due to the low biomass present and potential contaminations of the sequenced samples (Lauder *et al*. 2016).

Maternal administration of probiotics is another source of beneficial bacteria in the infant gut. In a small cohort study with six mother–infant dyads, all four infants delivered vaginally and in one of two C-section-born infants, *Lactobacillus rhamnosus* GG was present in infant faecal samples at 1 and 6 months of age when mothers were taking *L. rhamnosus* GG during late pregnancy (Schultz *et al*. 2004). With a larger cohort, *L. rhamnosus* GG was found in maternal (74 of 116 subjects at 3 months after delivery) and infant (50 of 129 subjects at 10 days; 56 of 122 at 3 months) stool samples following intake by the mother from 36 weeks of gestation up to 3 months postnatally (Dotterud *et al*. 2015).

### The infant gut virome

The infant gut virome, which includes eukaryotic and prokaryotic viruses (bacteriophage), has not been as intensively studied as the bacterial portion of the infant gut microbiota. One of the main difficulties in analysis of the infant gut virome is that a large number of sequences cannot be assigned to a taxonomic group (Reyes *et al*. 2015). The diversity of the infant gut virome is lower than that of adults (Breitbart *et al*. 2008; Pannaraj *et al*. 2018), but recent studies have shown that similar to bacteria, the infant gut virome is dynamic and changes throughout infancy, including an increase in alpha diversity (Reyes *et al*. 2015). In an early study of the infant gut virome 1 week after birth, it was found that more than half the viral genotypes present in infant stool could no longer be detected just 1 week later (Breitbart *et al*. 2008). In terms of alpha diversity, bacteriophage decrease from birth to 24 months, but eukaryotic species richness increases (Lim *et al*. 2015). In the first months after birth, Caudovirales are predominant, followed by a shift to Microviridae at 24 months, a similar time to the shift of the infant microbiota towards that of an adult. Eukaryotic viruses are low in abundance in early life (1 month) but increase thereafter, with Anelloviruses being dominant (Lim *et al*. 2015). In a second study of 20 twin pairs, the Anelloviridae, which targets eukaryotic hosts, were highly abundant until 15 to 18 months, after which it decreased (Reyes *et al*. 2015). In terms of bacteriophage, their abundance and diversity are inversely proportional to that of the bacterial population in the infant gut. In twins, the microbiota shifted from high bacteriophage-low bacterial diversity in the first month to high bacterial diversity-low bacteriophage by 2 years of age. The authors suggested that the low bacterial diversity in the first month of life leads to a contraction of the bacteriophage population, which subsequently allows the bacterial population to flourish (Lim *et al*. 2015). Pannaraj *et al*. (2018) found shared viruses between breast milk and corresponding infant stool in the first week of life, suggestive of vertical transmission.

The Siphoviridae family of the Caudovirales order includes bacteriophages that target bifidobacteria (Lugli *et al*. 2016). When looking at bifidobacterial prophage, ‘bifido(pro)phages’, it was found that bifidobacterial species such as *B. longum* that were high in relative abundance at a certain sampling point were then decreased at the next time point, which corresponded to an increase in the relative abundance in the corresponding bifido(pro)phage. Metatransriptomic analysis confirmed that the prophage were actively replicating and killing their hosts (Lugli *et al*. 2016). Duranti *et al*. (2017) later proved that such bifido(pro)phages could be vertically transmitted from mother to infant. A *B. longum* phage was identified in a mother's breast milk and the corresponding infant stool sample at two separate time points. A second *B. longum* phage was identified in a mother's faecal sample and in the corresponding faecal sample of the infant (Duranti *et al*. 2017).

The *Bacteroides* genus is also targeted by members of the gut virome. The Alpavirinae subfamily of the Microviridae is associated with the *Bacteroides* genus (Krupovic and Forterre 2011), while crAssphage, a Podoviridae bacteriophage first identified in 2014 has also been identified in the infant gut virome and predates *Bacteroides* (Dutilh *et al*. 2014; Lim *et al*. 2015; McCann *et al*. 2018).

### The infant gut mycobiome

The gut mycobiota encompasses all the fungi present in the gastrointestinal tract. Similar to the virome, research into the infant gut mycobiota has gained traction in recent years. The fungal population in the infant gut tends to have a low biomass, which makes sequencing technologies such as shotgun sequencing difficult. Instead, amplicon-based techniques using the 18S or 23S rRNA gene sequence or internal transcribed spacer regions or culture-dependent approaches are utilised (Ward, Knights and Gale 2017).

In a study of faecal samples of 11 infants whose age range was not defined, *Candida albicans* was the dominant fungal species, followed by *Candida parapsilosis*, *Candida krusei* and *Leptosphaerulina* (Heisel *et al*. 2015). In a separate study of 111 participants that included 8 infants under 2 years of age, infants and children were found to have a higher fungal richness compared with adults. *Penicillium*, *Aspergillus*, *Candida*, *Debaryomyces*, *Malassezia*, *Ascomycota*, *Eurotiomycetes*, *Tremellomycetes*, *Nectriaceae* and *Trichosporon* were the dominant genera, with *Penicillium* being significantly more abundant in infants compared with adults (Strati *et al*. 2016). In contrast, in a study of 70 healthy control infants at 3 months of age, *Saccharomycetales*, *Penecillium* and *Aspergillus* were the dominant genera (Arrieta *et al*. 2018).

*Candida* spp., which are highly abundant in the maternal vaginal and skin microbiota, have also been shown to be vertically transmitted between maternal and corresponding infant faecal samples (Bliss *et al*. 2008).

### The sporobiota in the infant gut

An endospore is a tough, dormant structure formed by certain types of bacteria, typically members of the phylum Firmicutes (Clostridiaceae and Lachnospiraceae families), which allows for greater resistance to otherwise inhospitable conditions such as extremes in temperature, oxygen, antibiotic exposure, UV radiation and nutrient and water deprivation. Such characteristics have led to challenges in health and disease, as the ability to form spores is linked to pathology, including persistent, chronic infection, resistance to antibiotics, relapses and spread of antimicrobial-resistance genes. Due to the significance of spore formers to human health and disease, it has been suggested that they should be considered as a separate grouping in microbiota studies. The term sporobiota has been suggested to cover the entirety of spore-forming bacteria in a population, while the term sporobiome has been used to define a collection of genomes of spore-forming bacteria related to a particular niche (Tetz and Tetz 2017).

The abundance of spore formers in the human gut microbiota is considered to be underrepresented in many metagenomic studies, for reasons including the resistance of endospores to traditional DNA isolation techniques (Filippidou *et al*. 2015), the high similarity between the 16S rRNA and housekeeping genes of otherwise unrelated spore formers (Wang *et al*. 2007) and the fact that spore formers tend to have larger genomes, resulting in fewer reads per gene per taxon (Galperin *et al*. 2012; Tetz and Tetz 2017). However, a study of the culturable bacteria of the adult gut microbiota found that 60% of the commensal bacteria in the gut are spore formers and 30% of the overall gut microbiota. Spore formers were found in several gut-associated families, including Lachnospiraceae, Ruminococcaceae and Clostridiaceae (Browne *et al*. 2016).

Spores are specialised for host–host transmission, which makes them ideal for colonisation of the developing infant gut microbiota (Tetz and Tetz 2017). The ability to form spores allows certain bacteria to colonise the infant gut early in low numbers and wait until conditions become more favourable, at which point they enter into a vegetative state and begin to multiply (Avershina *et al*. 2016).

Studies have also found that the *Clostridium* genus is present at low relative abundance (<1%) in breast milk (Jost *et al*. 2013; Murphy *et al*. 2017). For this reason, it is perhaps not surprising that studies have found a higher level of spore formers in formula-fed infants. In a study of 98 mother–infant pairs in Sweden, formula-fed infants had elevated levels of *Clostridioides difficile* in comparison to those who were breast-fed. Even in breastfed infants, the cessation of breastfeeding at 12 months also led to an increase in levels of *Clostridium* (Bäckhed *et al*. 2015). Another study of 107 mother–infant pairs in the United States found that the Erysipelotrichaceae family was prominent among non-exclusively breastfed infants (Pannaraj *et al*. 2017).

## INFLUENCES OF PERINATAL FACTORS ON MICROBIOTA DEVELOPMENT AND MATURATION IN INFANTS

### Maternal nutrition and gut microbiota

**Figure 1.**
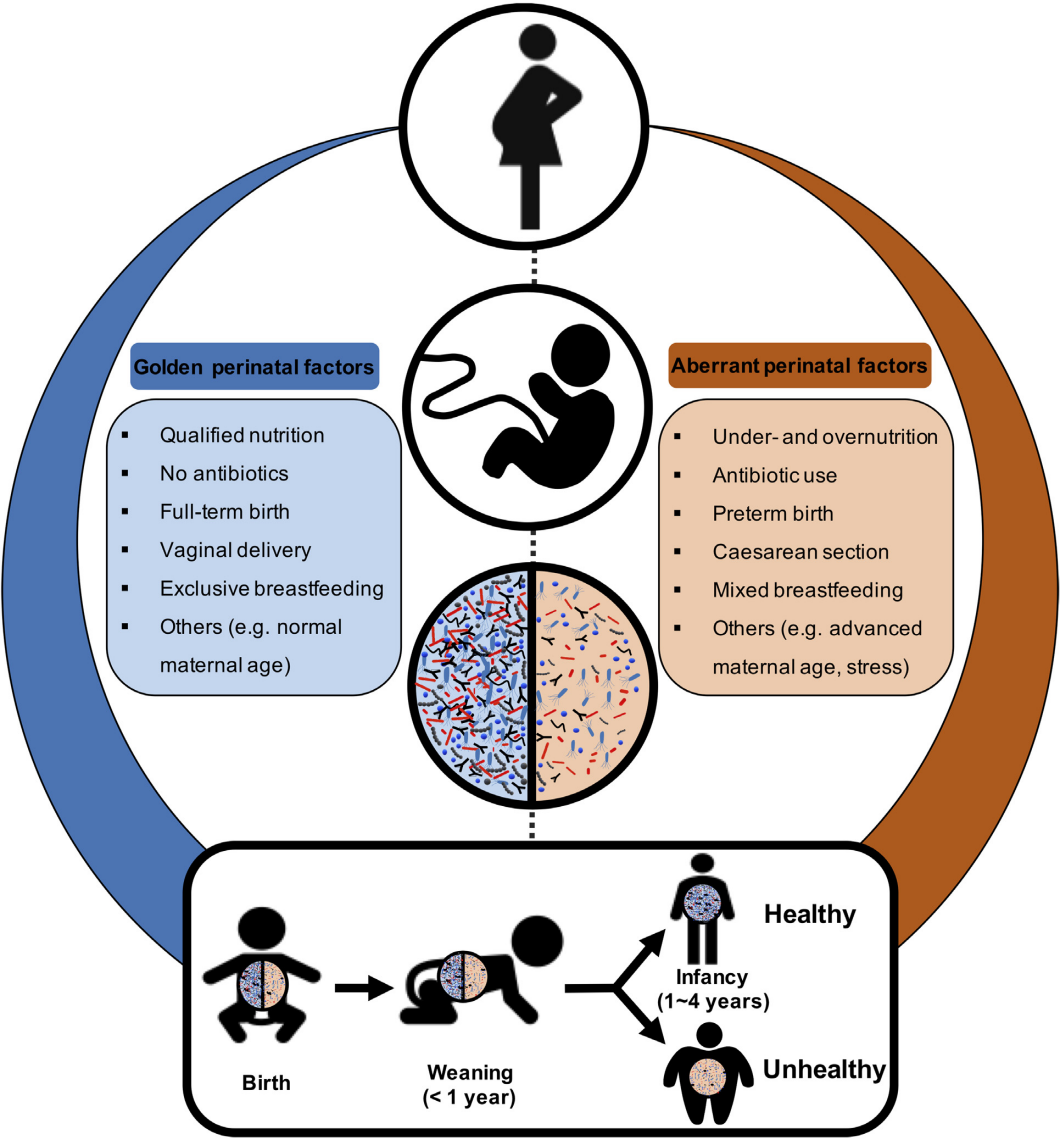
Perinatal factors impact the health and development of offspring through modulating the foetal growth, and the gut microbiota in both mothers and infants, which leaves a lasting impression beyond the birth and weaning period until childhood and even adulthood.

Maternal nutrient intakes during pregnancy and lactation have been linked to the acquisition and development of gut microbiota in the offspring (Chu *et al*. 2016b) (Fig. 1). In a human cohort with mother–infant dyads, where the mothers had a high-fat diet (>40%) within the third trimester of pregnancy based on a dietary questionnaire. An enrichment of species of *Lactococcus*, *Granulicatella* and *Enterococcus*, and a depletion of *Bacteroides*, *Sutterella*, *Parabacteroides* and *Comamonas* were observed in the gut microbiota of infants born to a maternal high-fat gestational diet, and this influence persisted to 6 weeks of age (Chu *et al*. 2016a). In addition, maternal fish and seafood intake during pregnancy increased the abundance of genus *Streptococcus* and decreased *Bacteroides uniformis* in infant gut at 6 weeks of age. Maternal dairy intake was positively associated with *Clostridium neonatale*, *Clostridium butyricum* and *Staphylococcus*, but decreased the abundance of Lachnospiraceae family. The abundance of genus *Bifidobacterium* was negatively associated with maternal fruit intake (Lundgren *et al*. 2018). The study additionally illustrated that this intergenerational nutritional influence on the gut microbiota of infants was in a delivery mode-dependent manner (Lundgren *et al*. 2018). In a non-human primate model [Japanese macaque (*Macaca fuscata*)], a maternal high-fat diet during pregnancy and lactation persistently shaped the gut microbiota in the offspring with reduced abundance of *Camplyobacter* species, and this impact could persist until adulthood at 1 year of age (Ma *et al*. 2014).

The underlying biological mechanisms regarding how maternal gestational diet modifies the offspring gut microbiota are not entirely understood. During pregnancy, it is known that the molecular transfer of nutrients from mother to foetus is of critical importance for early life immune development (Macpherson, de Agüero and Ganal-Vonarburg 2017). Alternation in maternal diets may disrupt this process, and thus interfere with the interactions of the host immune system with early-life microbiota. Considering the microbial mother-to-infant transmission at delivery and/or postpartum (Ferretti *et al*. 2018; Yassour *et al*. 2018; Wang *et al*. 2020), the maternal microbiota altered by diet could be transmitted to the offspring. Thus, further studies recording the information of maternal gestational diet, and sampling maternal and infant microbiota are warranted to address these knowledge gaps.

### Maternal antibiotic use and gut microbiota

Antibiotic treatment remains the major therapeutic strategy for many infectious diseases, however, increasing concerns have arisen due to its double-edged sword effect, in particular, the resulting risk of antibiotic resistance in the disease-causing microbes but also in beneficial commensal microbes (Fig. 1). Avoiding antibiotic use during the perinatal period is recommended when it is not mandatory, as increasing studies have identified associations between antibiotic use and the short- and long-term alternations of the microbiota and the health of the offspring.

The typical application of antibiotic administration during labour (referred to as intrapartum antibiotic prophylaxis, IAP) is for group B *Streptococcus* (GBS) positive women to prevent neonatal GBS infection and subsequent sepsis associated deaths (Al-Taiar *et al*. 2011). Meanwhile, changes of microbiota in newborns caused by IAP treatment have been disclosed by various approaches including routine microbiological culturing, molecular methods and next-generation sequencing in humans and animals. Using a molecular method of real-time polymerase chain reaction (PCR) for specific groups of bacteria, the absolute abundance of the *Bifidobacterium* genus in the gut microbiota of full-term and vaginally delivered newborns within 1 week after birth whose mothers were subjected to IAP treatment was decreased compared with newborns of mothers without antibiotics administered (Aloisio *et al*. 2014). Later, the author applied 16S amplicon sequencing to examine the effects of IAP on the gut microbiota of newborns in full-term and vaginally delivered newborns within 1 week after birth (Aloisio *et al*. 2016). In addition to confirmation of reduced bifidobacteria in newborns, the richness and diversity of the gut microbiota in newborns from the IAP group were decreased, with a lower abundance of Actinobacteria and Bacteroidetes as well as an overrepresentation of the Proteobacteria phylum and Enterobacteriaceae family (Aloisio *et al*. 2016). These findings were partially observed in other cohorts (Mazzola *et al*. 2016; Nogacka *et al*. 2017; Tapiainen *et al*. 2019). Within the first 6 months of life, differences in gut microbiota induced by IAP decreased gradually (Mazzola *et al*. 2016; Nogacka *et al*. 2017; Tapiainen *et al*. 2019). Bifidobacteria appeared to recover with a significant increase in the abundance similar to infants born normally but not for the Enterobacteriaceae family (Mazzola *et al*. 2016). Importantly, effects of postnatal antibiotic treatment to infants were comparable to that of IAP (Tapiainen *et al*. 2019).

Along with changes to the gut microbiota, enriched antimicrobial resistance genes (ARGs) in infants exposed to perinatal antibiotics have been noticed (Pärnänen *et al*. 2018; Tapiainen *et al*. 2019). These increased ARGs might result from, but are not limited to mother-to-infant transfer of maternal ARGs as there was a significant increase in the abundance of ARGs in the infants compared with their paired mothers (Pärnänen *et al*. 2018; Tapiainen *et al*. 2019), indicating that infants were at a high risk for the selection of antibiotic resistant strains once exposed to antibiotics, possibly due to the lesser richness or simpler composition of the gut microbiota in comparison to the adults (Pärnänen *et al*. 2018; Tapiainen *et al*. 2019).

The precise mechanism underlying the effects of maternal antibiotic intervention on the microbiota of offspring is still unknown. The response to antibiotics varies among species and the type and number of antibiotics administered. For example, the absolute abundance of *Lactobacilllus*, *B. fragilis*, *C. difficile* and *Escherichia coli* did not show any significant variation except for bifidobacteria in newborns of mothers exposed to IAP (Aloisio *et al*. 2014). The oral microbiota of offspring whose mothers received a cocktail of antibiotics clustered separately from those of mothers administered only a single antibiotic (Gomez-Arango *et al*. 2017). In addition, given that vertical transmission of microbes from mothers to infants is one of the critical sources for early-life microbiota (Ferretti *et al*. 2018; Yassour *et al*. 2018), perinatal antibiotic intervention in mothers may promote transmission of specific microbial strains to infants. Using a culture-dependent approach, a reduction of vaginal *Lactobacillus* transmission was observed from IAP-treated mothers to the infants’ oral cavity (Keski-Nisula *et al*. 2013), the result of which was later confirmed and expanded by 16S amplicon sequencing (Gomez-Arango *et al*. 2017). A high proportion (65%) of oral microbiota in newborns within 3 days after delivery was shared with maternal microbiota in the oral cavity; and maternal exposure to intrapartum antibiotics reduced the relative abundance of oral bacteria from families Micrococcaceae, Streptococcaceae, Gemellaceae and order Lactobacillales but increased the abundance of families Prevotellaceae, Bradyrhizobiaceae, Sphingomonadaceae, Comamonadaceae, Oxalobacteraceae and Neisseriaceae in the newborn (Gomez-Arango *et al*. 2017). Furthermore, perinatal antibiotic intervention can disrupt maternal microbiota, which may lead to an altered microbiota transferred to the offspring. This has been observed in a murine model (Miyoshi *et al*. 2017) but confirmation in humans still remains unexplored.

### Gestational age and microbiota

Prematurity strongly influences the initial gut microbiota in the newborn, and the subsequent trajectory of microbiota development and maturation (Fig.   1). Preterm infants are normally subjected to a microbiologically controlled environment (namely NICU) in the first few days of life or even longer depending on the newborn's health status. Exploring the development of the gut microbiota in the preterm infant is emerging as a critical research priority in the field of microbiology and paediatrics, due to the fact that perturbation of the gut microbiota during this key developmental window influences host physiology and disease risk (Groer *et al*. 2014). Up to now, our appreciation of gut microbiota in preterm infants is still relatively limited compared with full-term infants.

In the first few months, the richness of the gut microbiota colonising preterm infants accounted for only 10% of the bacterial species of the counterpart full-term infants (Gibson *et al*. 2016). The gut microbial composition in preterm infants was mainly comprised of microbes from classes of Bacilli, Gammaproteobacteria and Clostridia, accounting for >90% of gut microbiota, and each of the three classes represents the preponderance at different periods of life, with increasing proportions of Clostridia at the cost of Bacilli (La Rosa *et al*. 2014). In a large longitudinal study with 401 stool samples from 84 preterm infants aged from 1 week to 5 months, 6 bacterial species (*Enterococcus faecalis*, *Enterobacter cloacae*, *Staphylococcus epidermidis*, *E. coli*, *Klebsiella pneumoniae* and *Klebsiella oxytoca*) were consistently present in 99.8% samples from all preterm infants with high relative abundance (25–72%) (Gibson *et al*. 2016). The longitudinal pattern of gut microbiota development mentioned in La Rosa *et al*. (2014) was revealed to be driven primarily by species of *Klebsiella*, *Escherichia* and *Enterobacter* replacing *Enteroccoccus* and *Staphylococcus* as infants aged (Gibson *et al*. 2016). In addition, preterm infants have lower levels of *Bifidobacterium* and *Bacteroides* compared with full-term infants (Stewart *et al*. 2017; Chernikova *et al*. 2018). Sampling preterm infants at the early life stage is relatively undemanding, but there is a paucity of reports regarding the maturation of preterm infant gut microbiota in later life. A recent study following participants up to 4 years of age indicated that impact of gestational age at birth on the diversity and composition of gut microbiota in preterm children persisted at 1, 2 and 4 years, i.e. lower diversity, and discriminatory genera (*Lactobacillus*, *Streptococcus* and *Carnobacterium*) determined by linear discriminant analysis effect size for preterm children (Fouhy *et al*. 2019), indicating a delayed succession of microbial species in preterm infants (Arboleya *et al*. 2016).

The causes of preterm birth are complex (as discussed above), and a spectrum of vaginal microbiota is a significant contributor, particularly in women of African ancestry (Kindinger *et al*. 2017; Brown *et al*. 2018; Fettweis *et al*. 2019). The vaginal microbiota of women who gave preterm birth was characterised with low abundance of *Lactobacillus crispatus* and high levels of bacterial vaginosis-associated bacterium 1, *Sneathia amnii*, TM7-H1 and a group of *Prevotella* species (Fettweis *et al*. 2019). The preterm-associated bacteria generally expressed a series of proinflammatory cytokines into vaginal fluid, which were proposed to induce preterm birth (Fettweis *et al*. 2019). Combining these findings may provide a potential predictor for the risk of preterm birth early in pregnancy. However, causality still needs to be determined.

Developing strategies to prevent the adverse outcomes of preterm birth by intervening in the gut microbiota in early life is desirable. Feeding breast milk from own mothers or donors since the first days of life has been shown to stimulate the recovery and establishment of normal-like microbiota with gradually increased levels of bifidobacteria (Korpela *et al*. 2018; Parra-Llorca *et al*. 2018). In addition, supplementation of multispecies probiotics containing both *Lactobacillus* and *Bifidobacterium* species has been found to significantly reduce the risk of NEC in preterm infants (Kona and Matlock 2018).

### Mode of delivery and gut microbiota

Influences of mode of delivery on the acquisition and colonisation of microbiota across multiple body sites (gut, skin, nares and oral cavity) in infancy ranging from birth up to 4 years have been well documented (Dominguez-Bello *et al*. 2010; Chu *et al*. 2017; Fouhy *et al*. 2019) (Fig. 1). The much more diverse set of microbiota residing within the gut following C-section birth compared with the other body sites is of particular relevance to various disorders including allergies, obesity and inflammatory bowel disease (Tamburini *et al*. 2016). Thus, the majority of emerging evidence regarding the abnormal microbiota related to mode of delivery (C-section versus vaginal delivery) is from the gastrointestinal tract, and more studies are necessary to address the effect of C-section on the other microbial niches in the human body.

While most studies report the significant influence of C-section on the gut microbiota immediately after birth, there are some inconsistencies between studies. For example, the phylogenetic diversity, richness and evenness of microbiota from C-section newborn in meconium were greater compared with vaginally delivered newborns (Bokulich *et al*. 2016), but not in Chu *et al*. (2017), and even the opposite result was observed in a small Chinese cohort (Shi *et al*. 2018). This perhaps is attributable to exact sampling points, sampling procedure, cohort size, genomic DNA extraction and sequencing approach, and other environmental and population variables. The C-section infants acquired gut microbial species typically on the skin surface, dominated by genera of *Staphylococcus, Corynebacterium* and *Propionibacterium* (Dominguez-Bello *et al*. 2010). This could be due to the different first major microbial exposures for C-section-born compared with vaginally born infants, as passing through the birth canal may be critical for establishing a healthy microbiota early in life (Dominguez-Bello *et al*. 2010).

As newborns grow to 1 month old, mode of delivery continues to be the dominant factor over breastfeeding and surrounding environment that affect the colonisation of the gut microbiota (Wampach *et al*. 2018; Shao *et al*. 2019). In this period, infants delivered by C-section are enriched with species of *Enterococcus* (*E. faecalis*, *E. faecium*), *Staphylococcus* (*S. epidermis*, *S. saprophyticus*, *S. lugdunensis*, *S. aureus*), *Streptococcus* (*S. parasanguinis*, *S. australis*), *Klebsiella* (*K. oxytoca*, *K. pneumoniae*), *Enterobacter* (*E. cloacae*, *E. hormaechei*, *E. cancerogenus*) and *Clostridium* (*C. perfringens*), *Haemophilus* (*H. parainfluenzae*, *H. aegyptius*, *H. influenzae*, *H. haemolyticus*), *Veillonella* (*V. dispar*, *V. parvula*), which are commonly associated with skin, oral cavity and the hospital environment, and partially are opportunistic pathogens (Bäckhed *et al*. 2015; Shao *et al*. 2019).

As infants grow, the abundance of species of *Bacteroides* that were undetectable in newborns delivered by C-section increased for the first time to enable detection at 6 months of age (Yassour *et al*. 2016), but was still less than vaginally born infants; conversely, Clostridiales and Enterobacteriaceae were more abundant in C-section-born infants. This difference remained until 1 year of age (Bäckhed *et al*. 2015; Bokulich *et al*. 2016). However, although nearly all of C-section-born infants from different cohorts lack members of the *Bacteroides* genus in their gut microbiota in the first few months of life, this feature still could not be confined solely to this group of infants. In a Finnish cohort, a subset (7/35) of vaginally born infants also had low abundance of *Bacteroides*; however these infants together with C-section-born infants exhibited high abundance of *Bifidobacterium* species. Functional analysis revealed that *Bifidobacterium* mainly contributed to HMOs breakdown in the low-*Bacteroides* group replacing *Bacteroides* species that were the main contributor in all normally delivered infants (Yassour *et al*. 2016). In such a case, in addition to confirmation of this hypothesis with larger cohorts, identification of the dominant degrader(s) of HMOs in those C-section-born infants with low relative abundance of *Bifidobacterium* and *Bacteroides* need to be addressed. However, it has to be mentioned that these observations were detected with the relative abundance of the microbial community and the absolute changes of these species were unclear.

This microbiota similarity between infants born vaginally or by C-section gradually increased after the first year of life (Bokulich *et al*. 2016; Fouhy *et al*. 2019), suggesting that both microbial ecosystems underwent maturation, resembling the adult-like gut microbiota. At 2 years of age, the discriminatory genera could be still identified for vaginally born infants, namely *Parabacteroides* and *Ruminiclostridium*, while *Gordonibacter* and Lachnospiracheae NC2004 group were discriminative of C-section-born infants. By 4 years of age, there were no discriminatory genera for C-section-born infants (Fouhy *et al*. 2019). The clinical consequences of the long-lasting and large-scale perturbations of early-life gut microbiota remain to be investigated.

Given that antibiotics normally are administered during pregnancy to mothers who give birth by C-section to prevent the risk of infections, in order to discriminate influences between mode of delivery and antibiotics, C-sections were specially performed with mice model without the use of antibiotics in the perinatal period. The maturation of gut microbiota in C-section-born mice was almost stagnated compared with the vaginally born mice showing a progressive approximation in microbial maturation during the first 6 weeks of life. The diversity of the gut microbiota in vaginally born animals decreased with age from weaning, which was also observed in C-section-born mice with lower diversity at weaning. In addition, C-section-born mice at weaning were enriched with *Lactobacillus*, and Erysipelotrichaceae with underrepresented taxa of *Bacteroides*, Ruminococcaceae, Lachnospiraceae and Clostridiales (Martinez *et al*. 2017). The research with the same hypothesis in humans is warranted.

A number of studies have shown a correlation between C-section delivery and a higher abundance of the spore-forming *Clostridium* genus. In the INFANTMET study, the *Clostridium* genus was found to be more abundant at 1 week in C-section-born infants, both full term and preterm, as compared with vaginally born infants. However, at later samples, no significant difference was observed. Interestingly, the metabolomic analysis performed in this study revealed that bile acids were present in higher amounts in preterm urine, compared with full term. *Clostridioides difficile* spores germinate in response to primary bile salts (Shen 2015), although this study did not specify which bile salts were found (Hill *et al*. 2017). In a recent comprehensive study of 596 healthy full-term babies, the spore former *Clostridium perfringens* was also found to be enriched in the gut microbiota of C-section delivered infants during the first week of life (Shao *et al*. 2019). In a study of 13 C-section delivered infants in southern Spain, *Clostridium* was present in all time points, in all infants and adults. Two of the infants in this cohort were found to have *Clostridium* in their meconium samples, which were recovered at 100% identity with the corresponding maternal sample, indicative of vertical transmission from mother to infant, possibly *in utero* (Vallès *et al*. 2014)

### Breastfeeding and microbiota

The early diet of infants in the first few months is dominated by the breast milk from their own mother or donor, or formula where breast milk is unavailable, followed by the introduction of solid food (Fig. 1). The early feeding pattern including the extent of breastfeeding, the timing of solid food introduction and other provided dietary ingredients such as prebiotics, probiotics and symbiotics may result in different trajectories of gut microbiota development.

Breast milk harbours >700 bacterial species at concentrations of ∼1000 colony-forming units (CFUs)/ml, and thus breastfed infants ingest up to ∼800 000 bacteria daily (Le Doare *et al*. 2018). The origins of the human milk microbiota may stem from maternal skin and the oral cavity of the infants or possibly translocate from the maternal gut by the entero-mammary pathway, although this theory is still controversial and needs to be confirmed (Rodríguez 2014). The microbial profile of the breast milk can change over the course of lactation from colostrum, to transition and mature breast milk. Species of *Weissella* and *Leuconostoc*, followed by *Staphylococcus*, *Streptococcus* and *Lactococcus*, dominate the microbial community in colostrum (Cabrera-Rubio *et al*. 2012). Afterwards, genera of *Pseudomonas*, *Staphylococcus* and *Streptococcus* constitute the main members of a core set of microbial species in breast milk, while the other members vary across studies, possibly attributed to the different stages of lactation, maternal health status and delivery mode (Cabrera-Rubio *et al*. 2012; Jost *et al*. 2013; Khodayar-Pardo *et al*. 2014; Murphy *et al*. 2017).

A close relationship between the infant's gut microbiota and mother's breast milk microbiota has been established, and it is dependent on the infant’s age. During the first week of life, studies showed that the feeding pattern, either exclusive breastfeeding or non-exclusive breastfeeding, did not affect the microbiota in the newborn's gut (Bäckhed *et al*. 2015; Shao *et al*. 2019). As newborns grow, breastfeeding becomes the most important factor shaping the gut microbiota (Stewart *et al*. 2018). Within 6 months, exclusively breastfed infants had increased levels of species of *Lactobacillus* (*L. johnsonii*, *L.gasseri*, *L. paracasei*, *L. casei*), and *B. longum*. In contrast, the relative abundance of *Bacteroides*, *Eubacterium*, *Veillonella* and *Megasphaera* in non-exclusively breastfed infants increased (Bäckhed *et al*. 2015; Ho *et al*. 2018).

In general, microbial diversity is higher in non-exclusively breastfed than exclusively breastfed infants in early life (Ho *et al*. 2018) and during 12 to 24 months of life (Bokulich *et al*. 2016). The maturation of microbiota in infants fed exclusively by breast milk occurs later than in infants who received breast milk and formula, reflected by the predicted microbial age (Bäckhed *et al*. 2015; Ho *et al*. 2018). An increase in gut microbial age is related to a shorter duration of exclusively breastfeeding (Ho *et al*. 2018). Breast-fed dominant infants aged from 12 to 24 months were enriched with species of *Lactobacillus*, *Staphylococcus*, *Megasphaera* and Actinobacteria, while genera of Clostridiales and Proteobacteria were more abundant in formula-fed dominant infants (Bokulich *et al*. 2016).

The influence of feeding pattern depends on the other perinatal factors, such as mode of delivery. A study showed that the impact of breastfeeding on the gut microbiota in infants became significant only after the first week (Shao *et al*. 2019). The effect size of breastfeeding was still smaller than mode of delivery, and importantly breastfeeding did not impact the gut microbiota differently according to mode of delivery during the first month of life (Reyman *et al*. 2019). Infants born by C-section and fed by breast milk did not gain the comparable abundance of *Bifidobacterium* at 1 week of life compared with vaginally born infants fed by formula (Reyman *et al*. 2019). As infants age to around 8 months, the influence of breastfeeding was increasing and becoming comparable to the impact of mode of delivery (Shao *et al*. 2019). Conversely, results from the TEDDY cohort indicated that breastfeeding dominated the microbial development as measured from 3 to 14 months of life compared with the other perinatal factors including mode of delivery that significantly influences the gut microbiota of infants (Stewart *et al*. 2018). The contradictory results might be attributable to the statistical analysis methods, the heterogeneity among populations such as the breastfeeding cessation in practice, and the clinical structure of the involved participants (e.g. the ratio of C-section to vaginally born infants, the extent of exclusive breastfeeding) (Shao *et al*. 2019).

### Maternal stress and gut microbiota

With increasing evidence that the microbiota is one of the key regulators of the gut–brain axis (Cryan *et al*. 2019), influences of maternal stress during pregnancy on the offspring's psychological function and behaviour, and physical development and health have been linked to the maternal microbiota (Fig. 1). An altered maternal microbiota as a result of perinatal stress may be transmitted to the offspring, which impacts the maturation of an infant's immunity, and the hypothalamic-pituitary-adrenal axis (Zijlmans *et al*. 2015; Gensollen *et al*. 2016). Indeed, infants of mothers who suffered from high cumulative stress assessed by a combination of high reported stress and high cortisol concentrations during pregnancy had a higher microbial diversity, and were enriched in species of Proteobacteria (*Escherichia*, *Enterobacter*, *Serratia*) with lower abundances of lactic acid bacteria (*Lactobacillus*, *Lactoccus*, *Aerococcus*) and Actinobacteria (bifidobacteria, *Collinsella*, *Eggerthella*) during the first 4 months of life. The altered microbial colonisation pattern increased the risk of predisposing the infant to gastrointestinal symptoms and allergic reactions (Zijlmans *et al*. 2015). Later, as shown in mice, this early prenatal stress was proven to influence the acquisition and development of gut microbiota in a temporal and sex-specific manner via altering the maternal gut and vaginal microbiota (Jašarević *et al*. 2017), and lasted until adulthood in female mice (Gur *et al*. 2017), which needs to be confirmed further in humans.

## ASSOCIATION OF MICROBIOTA IN EARLY LIFE WITH HEALTH

As discussed above, a number of intrinsic and extrinsic perinatal factors can affect the composition of the infant gut microbiota. The next logical question is what is the effect of these changes, particularly in relation to infant health and development. The hygiene hypothesis, although somewhat controversial, claims that the increasing incidence of allergic and auto-immune diseases in western countries can be explained by changes in early microbial exposure as colonisation of the infant gut is considered a critical factor in training the immune system's reactions to microorganisms (Penders *et al*. 2014).

### Development of the immune system and risk of allergy

The gut microbiota plays a key role in the development and maturation of the infant immune system. Reduced diversity in the infant gut microbiota has been linked with a range of auto-immune diseases and allergies. Atopic disease relates to eczema (atopic dermatitis), allergic rhinitis (hay fever) or asthma and is generally defined by elevated levels of IgE in the serum. A lower bacterial diversity in early life (1 week to 1 month) has been linked with development of asthma and eczema later in childhood at 1 year (Ismail *et al*. 2012), one and half years (Wang *et al*. 2008) and 7 years of age (Abrahamsson *et al*. 2014). Lower bacterial richness at 3 months was also linked to an increased risk of food sensitisation at 1 year of age. However, there was no difference in bacterial richness or diversity between sensitised and non-sensitised infants at 1 year of age, which further highlights the importance of the development of the infant microbiota in the first months of life (Azad *et al*. 2015). A recent study also found that the numbers of bacterial cells by real-time PCR in faecal samples of infants with atopic dermatitis were lower than healthy controls at 6 months. Although this study did not find a compositional difference in gut microbiota between the two groups, at a functional level the relative abundance of microbial genes involved in oxidative phosphorylation (involving regulatory T cells activation) and nucleotide-binding oligomerisation domain (NOD)-like receptor signalling (being a function of sensing commensal microbiota and maintaining homeostasis) were higher in the control group (Lee *et al*. 2018).

Specific phyla and genera of bacteria have also been linked with increased susceptibility to allergies. A recent longitudinal study followed the development of the IgE-mediated allergies and compositional changes in the gut microbiota in 93 children from 4 months to 8 years. In children that developed allergies, *Bacteroides* were significantly underrepresented in early samples, which persisted to 8 years. *Coprococcus* and *Prevotella* were also underrepresented in allergic children. Interestingly and perhaps unexpectedly given their status as health-benefiting bacteria, bifidobacteria were enriched in allergic children compared to nonallergic children, along with OTUs from the Ruminococcaceae family. *Roseburia* and *Clostridium*, including *C. difficile*, were also consistently overrepresented in allergic children. This study hypothesised that the production of butyrate by *Coprococcus* plays a key role in preventing the development of allergic disease (Simonyté Sjödin *et al*. 2019). Butyrate is the preferred energy source of colonic epithelial cells while also playing a role in maintaining gut barrier function (Lopetuso *et al*. 2013).

Higher levels of Enterobacteriaceae and lower levels of Bacteroidaceae at 3 months of age were associated with an increased risk of food sensitisation (Azad *et al*. 2015). The KOALA study in the Netherlands identified a link between the presence of *C. difficile* and the development of atopic diseases such as asthma, eczema and sensitisation towards food allergies. This study identified not only C-section birth with increased risk of atopic diseases but also hospital birth. Vaginally born infants in hospital had an increased risk compared with those born at home, which correlated with increased colonisation by *C. difficile* (van Nimwegen *et al*. 2011).

Several studies have found that an underrepresentation of *Bacteroides* is linked with an increased risk of allergic disease. As described elsewhere in this review, this genus is significantly affected by mode of delivery, with a sustained decrease in relative abundance in C-section-born infants. C-section birth and the lower abundance of *Bacteroides* compared with vaginally born controls was associated with lower levels of Th1-associated chemokines in a study of 24 infants over the first 2 years of life, indicating a reduced Th1 immune response (Jakobsson *et al*. 2014). A low diversity in the *Bacteroides* genus at the first month of life was associated with IgE-mediated atopic eczema during the first 2 years (Abrahamsson *et al*. 2012). Another study found that the Bacteroidetes were 3-fold less abundant in infants with eczema at 18 months (Nylund *et al*. 2013). The *Bacteroides* genus was also found to be underrepresented in infants with IgE-mediated food allergies (Ling *et al*. 2014).

The presence of *Lactobacillus* and *Bifidobacterium* has been associated with a reduced susceptibility to allergic diseases (Sjögren *et al*. 2009a; Johansson *et al*. 2011). *Lactobacillus* strains can induce IL-12 and INF-*γ*, which results in the suppression of IgE (Shida *et al*. 1998). Increased bifidobacterial diversity is associated with increased IgA production and protection against allergy (Sjögren *et al*. 2009b).

### Diabetes

Type I Diabetes (T1D) is an auto-immune disorder that results from T cell-mediated destruction of the insulin-producing β cells of the pancreatic islets. Although there is a genetic element to the development of T1D, it has been suggested that pathogenesis may be influenced by the gut microbiota (Wen *et al*. 2008). In Eastern Europe, T1D is prevalent in Finnish and Estonian children. In a study of 33 infants from Finland and Estonia who were genetically at risk for the development of T1D, it was found that there was a 25% decrease in alpha diversity in T1D-diagnosed individuals compared with those who had not developed the disease. At genus level, *Ruminococcus* and *Streptococcus* were overrepresented in T1D-diagnosed infants but the differences were not significant (Kostic *et al*. 2015). Another study in Eastern Europe linked the prevalence of T1D in Finland and Estonia to a higher relative abundance of *Bacteroides* in the infant gut microbiota. In comparison, infants from Russia had a lower relative abundance of *Bacteroides* and a lower prevalence of T1D (Vatanen *et al*. 2016). These results correlated with a wider European study that also found an increased abundance of *Bacteroides* in children younger than 2.9 years who developed T1D (de Goffau *et al*. 2014). A transatlantic longitudinal study described changes in the functional potential of the microbiota, correlating a decrease in fermentation pathways and the production of short-chain fatty acids with an increased incidence of T1D development in infants genetically at risk (Vatanen *et al*. 2018).

### Obesity

Compositional changes in the infant gut microbiota have also been linked with a tendency towards obesity later in life. Obese adults typically have lower numbers of Bacteroidetes than normal-weight individuals (Kotzampassi, Giamarellos-Bourboulis and Stavrou 2014) and as described above C-section-born infants also lack this group. An early study using fluorescent in situ hybridization (FISH) with flow cytometry found that a microbiota population with an abundance of bifidobacteria and lower numbers of *Staphylococcus aureus* in early life (6 months) was shown to inversely correlate with obesity by the age of 7 (Kalliomäki *et al*. 2008). This was corroborated by a later study using 16S rRNA sequencing that found that high *Bifidobacterium* and *Collinsella* levels at 6 months of age corresponded with lower adiposity at 18 months. On the contrary, high levels of *Streptococcus* were associated with earlier gestational age and higher adiposity (Dogra *et al*. 2015).

### Probiotic intervention studies

Probiotics are defined as ‘live microorganisms which when administered in adequate amounts confer a health benefit on the host’. Due to the adverse effects attributed to perturbations in the infant gut microbiota described above, probiotic intervention studies have increased among the scientific and medical communities. The majority of such studies utilise strains of bifidobacteria and lactobacilli, due to their technical robustness and generally safe status, but overall results have been mixed.

*Lactobacillus rhamnosus* GG has been widely used in such studies. As mentioned above, this strain has been shown to transfer to the infant following use by the mother (Schultz *et al*. 2004; Dotterud *et al*. 2015). This probiotic strain was also used in an intervention study in infants who were at risk of developing asthma in Northern Europe. Administration of the strain daily from birth to 6 months resulted in a distinct community composition including an increased relative abundance of Lactobacillaceae and Bifidobacteriaceae. The community composition was also more stable in the high *L. rhamnosus* GG group that the authors speculated could be a protective mechanism against pathogen overgrowth (Cox *et al*. 2010). *Lactobacillus rhamnosus* GG was also used in a probiotic mixture containing *Lactobacillus acidophilus* La-5 and *Bifidobacterium animalis* subsp. *lactis* BB-12 to investigate if maternal intake of probiotics could decrease the risk of atopic disease in infants. The probiotic was found to reduce the incidence of atopic dermatitis but there was no effect on the composition of the gut microbiota of the infant (Dotterud *et al*. 2010). A later investigation of the data by the same group suggested that the effect of the probiotic is dependent on the intrinsic microbiota of the infant (Avershina *et al*. 2017). Supplementation with *B. longum*subsp. *infantis* EV001 was shown to reduce levels of faecal calprotectin in the first 2 months after birth (Henrick *et al*. 2019). High levels of faecal calprotectin are indicative of intestinal inflammation and an increased risk of atopic dermatitis and asthma later in life (Orivuori *et al*. 2015).

Prevention of atopic disease was also the primary objective of a longitudinal perinatal probiotic intervention study. In this study, pregnant mothers were given a capsule containing *L. rhamnosus* GG, *L. rhamnosus*, *B. breve* Bb99 and *Propionibacterium freudenreichii*ssp.*shermanii* DSM 7076 and after birth their infants received the same probiotic daily for the first 6 months of life. The prevalence of atopic disease was measured at 2, 5 and 13 years. After 5 and 13 years, it was found that there was no significant difference in the overall cohort between the probiotic and placebo groups. However, in the C-section delivered subgroup who received the probiotic, there was a significant reduction in allergic disease and eczema (Kallio *et al*. 2019). A similar study investigated the administration of probiotic mixtures to mothers with allergic disease, prenatally and postnatally. The probiotic mixtures were *L. rhamnosus* LPR and *B. longum* BL999 or *Lactobacillus paracasei* ST11 and *B. longum* BL999. After 24 months, the risk of eczema in the infants was significantly reduced in both probiotic groups as compared with the control group (Rautava *et al*. 2012).

One particular area that has produced positive results in probiotic intervention studies is the prevention of NEC in preterm infants. The *L. rhamnosus* GG strain mentioned above was shown to reduce the incidence of NEC when used in conjunction with bovine lactoferrin (Manzoni *et al*. 2014). Another *Lactobacillus* strain, *Lactobacillus reuteri* DSM17938, was similarly effective in reducing the incidence of NEC (Hunter *et al*. 2012). In a comprehensive meta-analysis of 24 separate clinical trials, it was found that probiotic supplementation significantly reduced the rate of severe NEC and mortality. The studies utilised *Lactobacillus*, *Bifidobacterium*, *Saccharomyces boulardii* or a mixture of bacterial strains (AlFaleh and Anabrees 2014). However, a more recent trial using *B. breve* BBG-001 found no significant difference between probiotic and placebo groups (Costeloe *et al*. 2016). In preterm infants, oral supplementation with a probiotic mixture of *B. bifidum* and *L. acidophilus* was shown to restore the composition of the microbiome closer to that of healthy full-term infants (Alcon-Giner *et al*. 2019).

## ‘MISSING MICROBES’ FROM PERINATAL PERIOD

Some microbes have been observed (as described above) to be absent or at very low levels under certain circumstances, such as *Bacteroides* for C-section, *Bifidobacterium* and *Bacteroides* for preterm infants, *Bifidobacterium* for short-term breastfeeding or without breastfeeding (referred to as ‘missing microbes’). However, it has to be kept in mind that those missing microbes might be still present but cannot be detected with current approaches. For example, the missing *Bacteroides* from C-section-born infants gain prevalence in a few months of life, but the source of the recovered *Bacteroides* and the underlying mechanisms are still unknown. Similar to genetic mutations and modifications, the missing microbes are able to ‘mutate’ the profile of the initial members of the gut microbiota, resulting in a different trajectory of microbial development and host health in life. To this end, future research exploring the missing microbes as the ‘next-generation probiotics’ should be considered by leveraging the advances in sequencing technology and metagenomic analyses, together with improvements in culture-dependent techniques (Browne *et al*. 2016; Lagier *et al*. 2018), for example, *Bacteroides* species whose effect, by their presence or absence, is clearly fundamental in infant health and development (Tan, Zhai and Chen 2019).

On the other side, as the same functions can be shared by different species, it is possible that the other species with increased abundance in the ecosystem compensate for the functions left open by the missing microbes (Yassour *et al*. 2016). Thus, future research must incorporate more than the composition of the microbial community, and characterisation at functional level will be essential to uncover the functional interactions between commensal microbiota with the clinical consequence for the host.

## CONCLUSIONS

Associations between maternal perinatal factors and the health of the offspring have been confirmed by cohorts of various sizes. The impaired growth and development of the foetus due to aberrant perinatal elements such as maternal malnutrition and antibiotics, in particular affecting infant immunity, is proposed to leave a negative impression on infant health in early and later life; although, the underlying mechanisms of action are largely unknown. However, the gut microbiota has been indicated to be highly sensitive to a number of perinatal factors and could provide the missing link. Indeed, the microbiota is increasingly recognised as a biological bond between mothers and infants. Given the vital contribution of the maternal microbiota to the early-life microbiota, and the relationship between early-life microbiota and subsequent adverse health outcomes, inheriting an altered maternal microbiota could be detrimental to the offspring. Direct evidence combining the perinatal factors, maternal and infant microbiota, and health outcomes of the offspring is beginning to emerge, where emphasis on the longer term maturation of the offspring will be essential. In parallel, the discovery of key microbes that respond specifically to different perinatal factors represents an obvious strategy in the exploration of next-generation probiotics for therapeutic interventions that maintain/improve the health of mothers and children to ensure a good start in life.

## References

[bib1] Aagaard K, Ma J, Antony KM et al. The placenta harbors a unique microbiome. Sci Transl Med. 2014;6:237ra65. 10.1126/scitranslmed.3008599 PMC4929217 24848255

[bib2] Abenhaim HA, Tulandi T, Wilchesky M et al. Effect of cesarean delivery on long-term risk of small bowel obstruction. Obstet Gynecol. 2018;131:354–9. 29324607 10.1097/AOG.0000000000002440

[bib4] Abrahamsson TR, Jakobsson HE, Andersson AF et al. Low diversity of the gut microbiota in infants with atopic eczema. J Allergy Clin Immunol. 2012;129:434–40. 22153774 10.1016/j.jaci.2011.10.025

[bib3] Abrahamsson TR, Jakobsson HE, Andersson AF et al. Low gut microbiota diversity in early infancy precedes asthma at school age. Clin Exp Allergy. 2014;44:842–50. 24330256 10.1111/cea.12253

[bib5] Ahern GJ, Hennessy A, Ryan CA et al. Advances in infant formula science. Annu Rev Food Sci Technol. 2019;10:75–102. 30908947 10.1146/annurev-food-081318-104308

[bib6] Al-Taiar A, Hammoud MS, Thalib L et al. Pattern and etiology of culture-proven early-onset neonatal sepsis: a five-year prospective study. Int J Infect Dis. 2011;15:e631–4. 21715207 10.1016/j.ijid.2011.05.004

[bib7] Alcon-Giner C, Dalby MJ, Caim S et al. Microbiota supplementation with *Bifidobacterium* and *Lactobacillus* modifies the preterm infant gut microbiota and metabolome. bioRxiv. 2019;698092. 10.1016/j.xcrm.2020.100077 PMC7453906 32904427

[bib8] AlFaleh K, Anabrees J Probiotics for prevention of necrotizing enterocolitis in preterm infants. Evid-Based Child Health. 2014;9:584–671. 25236307 10.1002/ebch.1976

[bib9] Aloisio I, Mazzola G, Corvaglia LT et al. Influence of intrapartum antibiotic prophylaxis against group B *Streptococcus* on the early newborn gut composition and evaluation of the anti-*Streptococcus* activity of *Bifidobacterium* strains. Appl Microbiol Biotechnol. 2014;98:6051–60. 24687755 10.1007/s00253-014-5712-9

[bib10] Aloisio I, Quagliariello A, De Fanti S et al. Evaluation of the effects of intrapartum antibiotic prophylaxis on newborn intestinal microbiota using a sequencing approach targeted to multi hypervariable 16S rDNA regions. Appl Microbiol Biotechnol. 2016;100:5537–46. 26971496 10.1007/s00253-016-7410-2

[bib11] Andreas NJ, Kampmann B, Le-Doare KM Human breast milk: a review on its composition and bioactivity. Early Hum Dev. 2015;91:629–35. 26375355 10.1016/j.earlhumdev.2015.08.013

[bib12] Arboleya S, Sánchez B, Solís G et al. Impact of prematurity and perinatal antibiotics on the developing intestinal microbiota: a functional inference study. Int J Mol Sci. 2016;17:649. 10.3390/ijms17050649 PMC4881475 27136545

[bib13] Arrieta M-C, Arévalo A, Stiemsma L et al. Associations between infant fungal and bacterial dysbiosis and childhood atopic wheeze in a nonindustrialized setting. J Allergy Clin Immunol. 2018;142:424–34.. e10. 29241587 10.1016/j.jaci.2017.08.041 PMC6075469

[bib14] Arumugam M, Raes J, Pelletier E et al. Enterotypes of the human gut microbiome. Nature. 2011;473:174–80. 21508958 10.1038/nature09944 PMC3728647

[bib15] Asnicar F, Manara S, Zolfo M et al. Studying vertical microbiome transmission from mothers to infants by strain-level metagenomic profiling. mSystems. 2017;2:e00164–16. 28144631 10.1128/mSystems.00164-16 PMC5264247

[bib16] Avershina E, Lundgård K, Sekelja M et al. Transition from infant- to adult-like gut microbiota. Environ Microbiol. 2016;18:2226–36. 26913851 10.1111/1462-2920.13248

[bib17] Avershina E, Rubio RC, Lundgård K et al. Effect of probiotics in prevention of atopic dermatitis is dependent on the intrinsic microbiota at early infancy. J Allergy Clin Immunol. 2017;139:1399–402.e8. 27931973 10.1016/j.jaci.2016.09.056

[bib18] Azad MB, Konya T, Guttman DS et al. Infant gut microbiota and food sensitization: associations in the first year of life. Clin Exp Allergy. 2015;45:632–43. 25599982 10.1111/cea.12487

[bib20] Bergström A, Skov TH, Bahl MI et al. Establishment of intestinal microbiota during early life: a longitudinal, explorative study of a large cohort of Danish infants. Appl Environ Microbiol. 2014;80:2889–900. 24584251 10.1128/AEM.00342-14 PMC3993305

[bib21] Betrán AP, Ye J, Moller A-B et al. The increasing trend in caesarean section rates: global, regional and national estimates: 1990–2014. PLoS One. 2016;11:e0148343. 26849801 10.1371/journal.pone.0148343 PMC4743929

[bib22] Bezold KY, Karjalainen MK, Hallman M et al. The genomics of preterm birth: from animal models to human studies. Genome Med. 2013;5:34. 23673148 10.1186/gm438 PMC3707062

[bib23] Bliss JM, Basavegowda KP, Watson WJ et al. Vertical and horizontal transmission of *Candida albicans* in very low birth weight infants using DNA fingerprinting techniques. Pediatr Infect Dis J. 2008;27:231–5. 18277930 10.1097/INF.0b013e31815bb69d

[bib24] Blumfield ML, Hure AJ, MacDonald-Wicks LK et al. Dietary balance during pregnancy is associated with fetal adiposity and fat distribution. Am J Clin Nutr. 2012;96:1032–41. 23034964 10.3945/ajcn.111.033241

[bib25] Bokulich NA, Chung J, Battaglia T et al. Antibiotics, birth mode, and diet shape microbiome maturation during early life. Sci Transl Med. 2016;8:343ra82. 10.1126/scitranslmed.aad7121 PMC5308924 27306664

[bib26] Breitbart M, Haynes M, Kelley S et al. Viral diversity and dynamics in an infant gut. Res Microbiol. 2008;159:367–73. 18541415 10.1016/j.resmic.2008.04.006

[bib28] Browne HP, Forster SC, Anonye BO et al. Culturing of ‘unculturable' human microbiota reveals novel taxa and extensive sporulation. Nature. 2016;533:543–6. 27144353 10.1038/nature17645 PMC4890681

[bib27] Brown RG, Marchesi JR, Lee YS et al. Vaginal dysbiosis increases risk of preterm fetal membrane rupture, neonatal sepsis and is exacerbated by erythromycin. BMC Med. 2018;16:9. 29361936 10.1186/s12916-017-0999-x PMC5782380

[bib19] Bäckhed F, Roswall J, Peng Y et al. Dynamics and stabilization of the human gut microbiome during the first year of life. Cell Host Microbe. 2015;17:690–703. 25974306 10.1016/j.chom.2015.04.004

[bib29] Cabrera-Rubio R, Collado MC, Laitinen K et al. The human milk microbiome changes over lactation and is shaped by maternal weight and mode of delivery. Am J Clin Nutr. 2012;96:544–51. 22836031 10.3945/ajcn.112.037382

[bib30] Cappelletti M, Della Bella S, Ferrazzi E et al. Inflammation and preterm birth. J Leukoc Biol. 2016;99:67–78. 26538528 10.1189/jlb.3MR0615-272RR

[bib31] Chen L-W, Tint M-T, Fortier MV et al. Maternal macronutrient intake during pregnancy is associated with neonatal abdominal adiposity: the Growing Up in Singapore Towards healthy Outcomes (GUSTO) study. J Nutr. 2016;146:1571–9. 27385763 10.3945/jn.116.230730 PMC4973884

[bib32] Chernikova DA, Madan JC, Housman ML et al. The premature infant gut microbiome during the first 6 weeks of life differs based on gestational maturity at birth. Pediatr Res. 2018;84:71–9. 29795209 10.1038/s41390-018-0022-z PMC6082716

[bib33] Chow AW, Jewesson PJ Pharmacokinetics and safety of antimicrobial agents during pregnancy. Rev Infect Dis. 1985;7:287–313. 3895351 10.1093/clinids/7.3.287

[bib34] Chu DM, Antony KM, Ma J et al. The early infant gut microbiome varies in association with a maternal high-fat diet. Genome Med. 2016a;8:77. 27503374 10.1186/s13073-016-0330-z PMC4977686

[bib35] Chu DM, Ma J, Prince AL et al. Maturation of the infant microbiome community structure and function across multiple body sites and in relation to mode of delivery. Nat Med. 2017;23:314–26. 28112736 10.1038/nm.4272 PMC5345907

[bib36] Chu DM, Meyer KM, Prince AL et al. Impact of maternal nutrition in pregnancy and lactation on offspring gut microbial composition and function. Gut Microbes. 2016b;7:459–70. 27686144 10.1080/19490976.2016.1241357 PMC5103658

[bib37] Claesson MJ, Wang Q, O'Sullivan O et al. Comparison of two next-generation sequencing technologies for resolving highly complex microbiota composition using tandem variable 16S rRNA gene regions. Nucleic Acids Res. 2010;38:e200. 20880993 10.1093/nar/gkq873 PMC3001100

[bib38] Collado MC, Rautava S, Aakko J et al. Human gut colonisation may be initiated *in utero* by distinct microbial communities in the placenta and amniotic fluid. Sci Rep. 2016;6:23129. 27001291 10.1038/srep23129 PMC4802384

[bib39] Costeloe K, Bowler U, Brocklehurst P et al. A randomised controlled trial of the probiotic *Bifidobacterium breve* BBG-001 in preterm babies to prevent sepsis, necrotising enterocolitis and death: the Probiotics in Preterm infantS (PiPS) trial. Health Technol Assess. 2016;20:1–194. 10.3310/hta20660 PMC5027379 27594381

[bib40] Coussons-Read ME, Lobel M, Carey JC et al. The occurrence of preterm delivery is linked to pregnancy-specific distress and elevated inflammatory markers across gestation. Brain Behav Immun. 2012;26:650–9. 22426431 10.1016/j.bbi.2012.02.009 PMC4462138

[bib41] Cox MJ, Huang YJ, Fujimura KE et al. *Lactobacillus casei*abundance is associated with profound shifts in the infant gut microbiome. PLoS One. 2010;5:e8745. 20090909 10.1371/journal.pone.0008745 PMC2807455

[bib42] Crume TL, Brinton JT, Shapiro A et al. Maternal dietary intake during pregnancy and offspring body composition: the Healthy Start Study. Am J Obstet Gynecol. 2016;215:609.e1–8. 27371352 10.1016/j.ajog.2016.06.035 PMC5571832

[bib43] Cryan JF, O'Riordan KJ, Cowan CS et al. The microbiota-gut-brain axis. Physiol Rev. 2019;99:1877–2013. 31460832 10.1152/physrev.00018.2018

[bib45] de Goffau MC, Fuentes S, van den Bogert B et al. Aberrant gut microbiota composition at the onset of type 1 diabetes in young children. Diabetologia. 2014;57:1569–77. 24930037 10.1007/s00125-014-3274-0

[bib46] Del Chierico F, Vernocchi P, Petrucca A et al. Phylogenetic and metabolic tracking of gut microbiota during perinatal development. PLoS One. 2015;10:e0137347. 26332837 10.1371/journal.pone.0137347 PMC4557834

[bib47] Devereux G, Litonjua AA, Turner SW et al. Maternal vitamin D intake during pregnancy and early childhood wheezing. Am J Clin Nutr. 2007;85:853–9. 17344509 10.1093/ajcn/85.3.853

[bib48] Dogra S, Sakwinska O, Soh S-E et al. Dynamics of infant gut microbiota are influenced by delivery mode and gestational duration and are associated with subsequent adiposity. mBio. 2015;6:e02419–14. 25650398 10.1128/mBio.02419-14 PMC4323417

[bib49] Dominguez-Bello MG, Costello EK, Contreras M et al. Delivery mode shapes the acquisition and structure of the initial microbiota across multiple body habitats in newborns. Proc Natl Acad Sci USA. 2010;107:11971–5. 20566857 10.1073/pnas.1002601107 PMC2900693

[bib50] Dotterud C, Storrø O, Johnsen R et al. Probiotics in pregnant women to prevent allergic disease: a randomized, double-blind trial. Br J Dermatol. 2010;163:616–23. 20545688 10.1111/j.1365-2133.2010.09889.x

[bib51] Dotterud CK, Avershina E, Sekelja M et al. Does maternal perinatal probiotic supplementation alter the intestinal microbiota of mother and child? J Pediatr Gastroenterol Nutr. 2015;61:200–7. 25782657 10.1097/MPG.0000000000000781

[bib52] Duranti S, Lugli GA, Mancabelli L et al. Maternal inheritance of bifidobacterial communities and bifidophages in infants through vertical transmission. Microbiome. 2017;5:66. 28651630 10.1186/s40168-017-0282-6 PMC5485682

[bib53] Dutilh BE, Cassman N, McNair K et al. A highly abundant bacteriophage discovered in the unknown sequences of human faecal metagenomes. Nat Commun. 2014;5:4498. 25058116 10.1038/ncomms5498 PMC4111155

[bib54] Egan M, Motherway MOC, Ventura M et al. Metabolism of sialic acid by *Bifidobacterium breve* UCC2003. Appl Environ Microbiol. 2014;80:4414–26. 24814790 10.1128/AEM.01114-14 PMC4068672

[bib55] Egan M, van Sinderen D Carbohydrate metabolism in Bifidobacteria. : Mattarelli P, Biavati B, Holzapfel WH*et al* (). The Bifidobacteria and Related Organisms: Biology, Taxonomy, Applications. London, UK: Academic Press, 2018, 145–64.

[bib56] Ferretti P, Pasolli E, Tett A et al. Mother-to-infant microbial transmission from different body sites shapes the developing infant gut microbiome. Cell Host Microbe. 2018;24:133–45.e5. 30001516 10.1016/j.chom.2018.06.005 PMC6716579

[bib57] Fettweis JM, Serrano MG, Brooks JP et al. The vaginal microbiome and preterm birth. Nat Med. 2019;25:1012–21. 31142849 10.1038/s41591-019-0450-2 PMC6750801

[bib58] Filippidou S, Junier T, Wunderlin T et al. Under-detection of endospore-forming Firmicutes in metagenomic data. Comput Struct Biotechnol J. 2015;13:299–306. 25973144 10.1016/j.csbj.2015.04.002 PMC4427659

[bib59] Finlay JE, Özaltin E, Canning D The association of maternal age with infant mortality, child anthropometric failure, diarrhoea and anaemia for first births: evidence from 55 low-and middle-income countries. BMJ Open. 2011;1:e000226. 10.1136/bmjopen-2011-000226 PMC3191600 22021886

[bib60] Fouhy F, Watkins C, Hill CJ et al. Perinatal factors affect the gut microbiota up to four years after birth. Nat Commun. 2019;10:1517. 30944304 10.1038/s41467-019-09252-4 PMC6447568

[bib61] Galperin MY, Mekhedov SL, Puigbo P et al. Genomic determinants of sporulation in *Bacilli* and Clostridia: towards the minimal set of sporulation-specific genes. Environ Microbiol. 2012;14:2870–90. 22882546 10.1111/j.1462-2920.2012.02841.x PMC3533761

[bib62] Ganal-Vonarburg SC, Hornef MW, Macpherson AJ Microbial–host molecular exchange and its functional consequences in early mammalian life. Science. 2020;368:604–7. 32381716 10.1126/science.aba0478

[bib63] Gensollen T, Iyer SS, Kasper DL et al. How colonization by microbiota in early life shapes the immune system. Science. 2016;352:539–44. 27126036 10.1126/science.aad9378 PMC5050524

[bib64] Gibbs CM, Wendt A, Peters S et al. The impact of early age at first childbirth on maternal and infant health. Paediatr Perinat Epidemiol. 2012;26:259–84. 22742615 10.1111/j.1365-3016.2012.01290.x PMC4562289

[bib65] Gibson MK, Wang B, Ahmadi S et al. Developmental dynamics of the preterm infant gut microbiota and antibiotic resistome. Nat Microbiol. 2016;1:16024. 27572443 10.1038/nmicrobiol.2016.24 PMC5031140

[bib66] Goldenberg RL, Culhane JF, Iams JD et al. Epidemiology and causes of preterm birth. Lancet. 2008;371:75–84. 18177778 10.1016/S0140-6736(08)60074-4 PMC7134569

[bib67] Gomez-Arango LF, Barrett HL, McIntyre HD et al. Antibiotic treatment at delivery shapes the initial oral microbiome in neonates. Sci Rep. 2017;7:43481. 28240736 10.1038/srep43481 PMC5378909

[bib68] Gomez-Gallego C, Garcia-Mantrana I, Salminen S et al. The human milk microbiome and factors influencing its composition and activity. Semin Fetal Neonatal Med. 2016;21:400–5. 27286644 10.1016/j.siny.2016.05.003

[bib69] Groer MW, Luciano AA, Dishaw LJ et al. Development of the preterm infant gut microbiome: a research priority. Microbiome. 2014;2:38. 25332768 10.1186/2049-2618-2-38 PMC4203464

[bib71] Gurol-Urganci I, Bou-Antoun S, Lim C et al. Impact of caesarean section on subsequent fertility: a systematic review and meta-analysis. Hum Reprod. 2013;28:1943–52. 23644593 10.1093/humrep/det130

[bib70] Gur TL, Shay L, Palkar AV et al. Prenatal stress affects placental cytokines and neurotrophins, commensal microbes, and anxiety-like behavior in adult female offspring. Brain Behav Immun. 2017;64:50–8. 28027927 10.1016/j.bbi.2016.12.021

[bib72] Heisel T, Podgorski H, Staley CM et al. Complementary amplicon-based genomic approaches for the study of fungal communities in humans. PLoS One. 2015;10:e0116705. 25706290 10.1371/journal.pone.0116705 PMC4338280

[bib73] Henrick BM, Chew S, Casaburi G et al. Colonization by *B. infantis* EVC001 modulates enteric inflammation in exclusively breastfed infants. Pediatr Res. 2019;86:749–57. 31443102 10.1038/s41390-019-0533-2 PMC6887859

[bib74] Hill CJ, Brown JR, Lynch DB et al. Effect of room temperature transport vials on DNA quality and phylogenetic composition of faecal microbiota of elderly adults and infants. Microbiome. 2016;4:19. 27160322 10.1186/s40168-016-0164-3 PMC4862223

[bib75] Hill CJ, Lynch DB, Murphy K et al. Evolution of gut microbiota composition from birth to 24 weeks in the INFANTMET Cohort. Microbiome. 2017;5:4. 28095889 10.1186/s40168-016-0213-y PMC5240274

[bib76] Ho NT, Li F, Lee-Sarwar KA et al. Meta-analysis of effects of exclusive breastfeeding on infant gut microbiota across populations. Nat Commun. 2018;9:4169. 30301893 10.1038/s41467-018-06473-x PMC6177445

[bib77] Hunter C, Dimaguila MAV, Gal P et al. Effect of routine probiotic, *Lactobacillus reuteri* DSM 17938, use on rates of necrotizing enterocolitis in neonates with birthweight < 1000 grams: a sequential analysis. BMC Pediatr. 2012;12:142. 22947597 10.1186/1471-2431-12-142 PMC3472183

[bib78] Ismail IH, Oppedisano F, Joseph SJ et al. Reduced gut microbial diversity in early life is associated with later development of eczema but not atopy in high-risk infants. Pediatr Allergy Immunol. 2012;23:674–81. 22831283 10.1111/j.1399-3038.2012.01328.x

[bib79] Jakobsson HE, Abrahamsson TR, Jenmalm MC et al. Decreased gut microbiota diversity, delayed Bacteroidetes colonisation and reduced Th1 responses in infants delivered by caesarean section. Gut. 2014;63:559–66. 23926244 10.1136/gutjnl-2012-303249

[bib81] Javaid MK, Crozier SR, Harvey NC et al. Maternal vitamin D status during pregnancy and childhood bone mass at age 9 years: a longitudinal study. Lancet. 2006;367:36–43. 16399151 10.1016/S0140-6736(06)67922-1

[bib80] Jašarević E, Howard CD, Misic AM et al. Stress during pregnancy alters temporal and spatial dynamics of the maternal and offspring microbiome in a sex-specific manner. Sci Rep. 2017;7:44182. 28266645 10.1038/srep44182 PMC5339804

[bib82] Johansson MA, Sjögren YM, Persson J-O et al. Early colonization with a group of Lactobacilli decreases the risk for allergy at five years of age despite allergic heredity. PLoS One. 2011;6:e23031. 21829685 10.1371/journal.pone.0023031 PMC3148229

[bib83] Jost T, Lacroix C, Braegger C et al. Assessment of bacterial diversity in breast milk using culture-dependent and culture-independent approaches. Br J Nutr. 2013;110:1253–62. 23507238 10.1017/S0007114513000597

[bib84] Jost T, Lacroix C, Braegger CP et al. New insights in gut microbiota establishment in healthy breast fed neonates. PLoS One. 2012;7:e44595. 22957008 10.1371/journal.pone.0044595 PMC3431319

[bib86] Kalliomäki M, Carmen Collado M, Salminen S et al. Early differences in fecal microbiota composition in children may predict overweight. Am J Clin Nutr. 2008;87:534–8. 18326589 10.1093/ajcn/87.3.534

[bib85] Kallio S, Kukkonen AK, Savilahti E et al. Perinatal probiotic intervention prevented allergic disease in a Caesarean-delivered subgroup at 13-year follow-up. Clin Exp Allergy. 2019;49:506–15. 30472801 10.1111/cea.13321

[bib87] Keag OE, Norman JE, Stock SJ Long-term risks and benefits associated with cesarean delivery for mother, baby, and subsequent pregnancies: systematic review and meta-analysis. PLoS Med. 2018;15:e1002494. 29360829 10.1371/journal.pmed.1002494 PMC5779640

[bib88] Keski-Nisula L, Kyynäräinen HR, Kärkkäinen U et al. Maternal intrapartum antibiotics and decreased vertical transmission of *Lactobacillus* to neonates during birth. Acta Paediatr. 2013;102:480–5. 23398392 10.1111/apa.12186

[bib89] Khodayar-Pardo P, Mira-Pascual L, Collado M et al. Impact of lactation stage, gestational age and mode of delivery on breast milk microbiota. J Perinatol. 2014;34:599–605. 24674981 10.1038/jp.2014.47

[bib90] Kindinger LM, Bennett PR, Lee YS et al. The interaction between vaginal microbiota, cervical length, and vaginal progesterone treatment for preterm birth risk. Microbiome. 2017;5:6. 28103952 10.1186/s40168-016-0223-9 PMC5244550

[bib91] Kona SK, Matlock DN Probiotics, prebiotics, and synbiotics for preterm neonates. NeoReviews. 2018;19:e654–63.

[bib92] Korpela K, Blakstad EW, Moltu SJ et al. Intestinal microbiota development and gestational age in preterm neonates. Sci Rep. 2018;8:2453. 29410448 10.1038/s41598-018-20827-x PMC5802739

[bib93] Kostic AD, Gevers D, Siljander H et al. The dynamics of the human infant gut microbiome in development and in progression toward type 1 diabetes. Cell Host Microbe. 2015;17:260–73. 25662751 10.1016/j.chom.2015.01.001 PMC4689191

[bib94] Kotzampassi K, Giamarellos-Bourboulis EJ, Stavrou G Obesity as a consequence of gut bacteria and diet interactions. ISRN Obes. 2014;2014:651895. 24977101 10.1155/2014/651895 PMC3963190

[bib95] Krupovic M, Forterre P Microviridae goes temperate: microvirus-related proviruses reside in the genomes of Bacteroidetes. PLoS One. 2011;6:e19893. 21572966 10.1371/journal.pone.0019893 PMC3091885

[bib96] Kuhle S, Tong O, Woolcott C Association between caesarean section and childhood obesity: a systematic review and meta-analysis. Obes Rev. 2015;16:295–303. 25752886 10.1111/obr.12267

[bib98] Lagier J-C, Dubourg G, Million M et al. Culturing the human microbiota and culturomics. Nat Rev Microbiol. 2018;16:540–50. 29937540 10.1038/s41579-018-0041-0

[bib97] La Rosa PS, Warner BB, Zhou Y et al. Patterned progression of bacterial populations in the premature infant gut. Proc Natl Acad Sci USA. 2014;111:12522–7. 25114261 10.1073/pnas.1409497111 PMC4151715

[bib99] Lauder AP, Roche AM, Sherrill-Mix S et al. Comparison of placenta samples with contamination controls does not provide evidence for a distinct placenta microbiota. Microbiome. 2016;4:29. 27338728 10.1186/s40168-016-0172-3 PMC4917942

[bib101] Lean SC, Derricott H, Jones RL et al. Advanced maternal age and adverse pregnancy outcomes: a systematic review and meta-analysis. PLoS One. 2017;12:e0186287. 29040334 10.1371/journal.pone.0186287 PMC5645107

[bib100] Le Doare K, Holder B, Bassett A et al. Mother's milk: a purposeful contribution to the development of the infant microbiota and immunity. Front Immunol. 2018;9:361. 29599768 10.3389/fimmu.2018.00361 PMC5863526

[bib102] Lee M-J, Kang M-J, Lee S-Y et al. Perturbations of gut microbiome genes in infants with atopic dermatitis according to feeding type. J Allergy Clin Immunol. 2018;141:1310–9. 29339259 10.1016/j.jaci.2017.11.045

[bib103] Lewis A, Austin E, Galbally M Prenatal maternal mental health and fetal growth restriction: a systematic review. J Dev Orig Health Dis. 2016;7:416–28. 26983652 10.1017/S2040174416000076

[bib104] Li H, Zhou Y, Liu J The impact of cesarean section on offspring overweight and obesity: a systematic review and meta-analysis. Int J Obes. 2013;37:893–9. 10.1038/ijo.2012.195 23207407

[bib105] Lim ES, Zhou Y, Zhao G et al. Early life dynamics of the human gut virome and bacterial microbiome in infants. Nat Med. 2015;21:1228–34. 26366711 10.1038/nm.3950 PMC4710368

[bib106] Ling Z, Li Z, Liu X et al. Altered fecal microbiota composition associated with food allergy in infants. Appl Environ Microbiol. 2014;80:2546–54. 24532064 10.1128/AEM.00003-14 PMC3993190

[bib107] Londero AP, Rossetti E, Pittini C et al. Maternal age and the risk of adverse pregnancy outcomes: a retrospective cohort study. BMC Pregnancy Childbirth. 2019;19:261. 31337350 10.1186/s12884-019-2400-x PMC6651936

[bib108] Lopetuso LR, Scaldaferri F, Petito V et al. Commensal Clostridia: leading players in the maintenance of gut homeostasis. Gut Pathog. 2013;5:23. 23941657 10.1186/1757-4749-5-23 PMC3751348

[bib109] Lugli GA, Milani C, Turroni F et al. Prophages of the genus *Bifidobacterium* as modulating agents of the infant gut microbiota. Environ Microbiol. 2016;18:2196–213. 26627180 10.1111/1462-2920.13154

[bib110] Lundgren SN, Madan JC, Emond JA et al. Maternal diet during pregnancy is related with the infant stool microbiome in a delivery mode-dependent manner. Microbiome. 2018;6:109. 29973274 10.1186/s40168-018-0490-8 PMC6033232

[bib112] Macpherson AJ, de Agüero MG, Ganal-Vonarburg SC How nutrition and the maternal microbiota shape the neonatal immune system. Nat Rev Immunol. 2017;17:508–17. 28604736 10.1038/nri.2017.58

[bib111] Ma J, Prince AL, Bader D et al. High-fat maternal diet during pregnancy persistently alters the offspring microbiome in a primate model. Nat Commun. 2014;5:3889. 24846660 10.1038/ncomms4889 PMC4078997

[bib113] Manzoni P, Meyer M, Stolfi I et al. Bovine lactoferrin supplementation for prevention of necrotizing enterocolitis in very-low-birth-weight neonates: a randomized clinical trial. Early Hum Dev. 2014;90:S60–5. 24709463 10.1016/S0378-3782(14)70020-9

[bib114] Marcobal A, Barboza M, Sonnenburg ED et al. Bacteroides in the infant gut consume milk oligosaccharides via mucus-utilization pathways. Cell Host Microbe. 2011;10:507–14. 22036470 10.1016/j.chom.2011.10.007 PMC3227561

[bib115] Martinez KA, Devlin JC, Lacher CR et al. Increased weight gain by C-section: functional significance of the primordial microbiome. Sci Adv. 2017;3:eaao1874. 29026883 10.1126/sciadv.aao1874 PMC5636202

[bib116] Mazzola G, Murphy K, Ross RP et al. Early gut microbiota perturbations following intrapartum antibiotic prophylaxis to prevent group B streptococcal disease. PLoS One. 2016;11:e0157527. 27332552 10.1371/journal.pone.0157527 PMC4917232

[bib117] McCann A, Ryan FJ, Stockdale SR et al. Viromes of one year old infants reveal the impact of birth mode on microbiome diversity. PeerJ. 2018;6:e4694. 29761040 10.7717/peerj.4694 PMC5944432

[bib118] Mitchell AA, Gilboa SM, Werler MM et al. Medication use during pregnancy, with particular focus on prescription drugs: 1976–2008. Am J Obstet Gynecol. 2011;205:51.e1–8. 21514558 10.1016/j.ajog.2011.02.029 PMC3793635

[bib119] Miyoshi J, Bobe AM, Miyoshi S et al. Peripartum antibiotics promote gut dysbiosis, loss of immune tolerance, and inflammatory bowel disease in genetically prone offspring. Cell Rep. 2017;20:491–504. 28700948 10.1016/j.celrep.2017.06.060 PMC5667669

[bib120] Morgan DJ Drug disposition in mother and foetus. Clin Exp Pharmacol Physiol. 1997;24:869–73. 9363372 10.1111/j.1440-1681.1997.tb02707.x

[bib121] Mueller NT, Whyatt R, Hoepner L et al. Prenatal exposure to antibiotics, cesarean section and risk of childhood obesity. Int J Obes. 2015;39:665–70. 10.1038/ijo.2014.180 PMC4390478 25298276

[bib122] Murphy K, Curley D, O'Callaghan TF et al. The composition of human milk and infant faecal microbiota over the first three months of life: a pilot study. Sci Rep. 2017;7:40597. 28094284 10.1038/srep40597 PMC5240090

[bib123] Nakayama J, Kobayashi T, Tanaka S et al. Aberrant structures of fecal bacterial community in allergic infants profiled by 16S rRNA gene pyrosequencing. FEMS Immunol Med Microbiol. 2011;63:397–406. 22029688 10.1111/j.1574-695X.2011.00872.x

[bib124] Neuman H, Forsythe P, Uzan A et al. Antibiotics in early life: dysbiosis and the damage done. FEMS Microbiol Rev. 2018;42:489–99. 29945240 10.1093/femsre/fuy018

[bib125] Nogacka A, Salazar N, Suárez M et al. Impact of intrapartum antimicrobial prophylaxis upon the intestinal microbiota and the prevalence of antibiotic resistance genes in vaginally delivered full-term neonates. Microbiome. 2017;5:93. 28789705 10.1186/s40168-017-0313-3 PMC5549288

[bib126] Nylund L, Satokari R, Nikkilä J et al. Microarray analysis reveals marked intestinal microbiota aberrancy in infants having eczema compared to healthy children in at-risk for atopic disease. BMC Microbiol. 2013;13:12. 23339708 10.1186/1471-2180-13-12 PMC3563445

[bib127] Orivuori L, Mustonen K, de Goffau M et al. High level of fecal calprotectin at age 2 months as a marker of intestinal inflammation predicts atopic dermatitis and asthma by age 6. Clin Exp Allergy. 2015;45:928–39. 25758537 10.1111/cea.12522

[bib128] Örtqvist AK, Lundholm C, Halfvarson J et al. Fetal and early life antibiotics exposure and very early onset inflammatory bowel disease: a population-based study. Gut. 2019;68:218–25. 29321166 10.1136/gutjnl-2017-314352

[bib129] Pannaraj PS, Li F, Cerini C et al. Association between breast milk bacterial communities and establishment and development of the infant gut microbiome. JAMA Pediatr. 2017;171:647–54. 28492938 10.1001/jamapediatrics.2017.0378 PMC5710346

[bib130] Pannaraj PS, Ly M, Cerini C et al. Shared and distinct features of human milk and infant stool viromes. Front Microbiol. 2018;9:1162. 29910789 10.3389/fmicb.2018.01162 PMC5992295

[bib132] Parra-Llorca A, Gormaz M, Alcántara C et al. Preterm gut microbiome depending on feeding type: significance of donor human milk. Front Microbiol. 2018;9:1376. 29997594 10.3389/fmicb.2018.01376 PMC6030370

[bib133] Penders J, Gerhold K, Thijs C et al. New insights into the hygiene hypothesis in allergic diseases: mediation of sibling and birth mode effects by the gut microbiota. Gut Microbes. 2014;5:239–44. 24637604 10.4161/gmic.27905 PMC4063851

[bib134] Peters LL, Thornton C, de Jonge A et al. The effect of medical and operative birth interventions on child health outcomes in the first 28 days and up to 5 years of age: a linked data population-based cohort study. Birth. 2018;45:347–57. 29577380 10.1111/birt.12348 PMC6282837

[bib135] Purisch SE, Gyamfi-Bannerman C Epidemiology of preterm birth. Semin Perinatol. 2017;41:387–91. 28865982 10.1053/j.semperi.2017.07.009

[bib131] Pärnänen K, Karkman A, Hultman J et al. Maternal gut and breast milk microbiota affect infant gut antibiotic resistome and mobile genetic elements. Nat Commun. 2018;9:3891. 30250208 10.1038/s41467-018-06393-w PMC6155145

[bib136] Rautava S, Kainonen E, Salminen S et al. Maternal probiotic supplementation during pregnancy and breast-feeding reduces the risk of eczema in the infant. J Allergy Clin Immunol. 2012;130:1355–60. 23083673 10.1016/j.jaci.2012.09.003

[bib137] Reali A, Ximenes A, Cuzzolin L et al. Antibiotic therapy in pregnancy and lactation. J Chemother. 2005;17:123–30. 15920896 10.1179/joc.2005.17.2.123

[bib138] Reyes A, Blanton LV, Cao S et al. Gut DNA viromes of Malawian twins discordant for severe acute malnutrition. Proc Natl Acad Sci USA. 2015;112:11941–6. 26351661 10.1073/pnas.1514285112 PMC4586842

[bib139] Reyman M, van Houten MA, van Baarle D et al. Impact of delivery mode-associated gut microbiota dynamics on health in the first year of life. Nat Commun. 2019;10:4997. 31676793 10.1038/s41467-019-13014-7 PMC6825150

[bib140] Rodríguez JM The origin of human milk bacteria: is there a bacterial entero-mammary pathway during late pregnancy and lactation? Adv Nutr. 2014;5:779–84. 25398740 10.3945/an.114.007229 PMC4224214

[bib141] Romero R, Dey SK, Fisher SJ Preterm labor: one syndrome, many causes. Science. 2014;345:760–5. 25124429 10.1126/science.1251816 PMC4191866

[bib142] Salone LR, Vann Jr WF, Dee DL Breastfeeding: an overview of oral and general health benefits. J Am Dent Assoc. 2013;144:143–51. 23372130 10.14219/jada.archive.2013.0093

[bib143] Sandall J, Tribe RM, Avery L et al. Short-term and long-term effects of caesarean section on the health of women and children. Lancet. 2018;392:1349–57. 30322585 10.1016/S0140-6736(18)31930-5

[bib144] Sawyer KM, Zunszain PA, Dazzan P et al. Intergenerational transmission of depression: clinical observations and molecular mechanisms. Mol Psychiatry. 2019;24:1157–77. 30283036 10.1038/s41380-018-0265-4

[bib145] Schultz M, Göttl C, Young RJ et al. Administration of oral probiotic bacteria to pregnant women causes temporary infantile colonization. J Pediatr Gastroenterol Nutr. 2004;38:293–7. 15076629 10.1097/00005176-200403000-00012

[bib146] Sela DA, Chapman J, Adeuya A et al. The genome sequence of *Bifidobacterium**longum* subsp. infantis reveals adaptations for milk utilization within the infant microbiome. Proc Natl Acad Sci USA. 2008;105:18964–9. 19033196 10.1073/pnas.0809584105 PMC2596198

[bib147] Shao Y, Forster SC, Tsaliki E et al. Stunted microbiota and opportunistic pathogen colonization in caesarean-section birth. Nature. 2019;574:117–21. 31534227 10.1038/s41586-019-1560-1 PMC6894937

[bib148] Shen A A gut odyssey: the impact of the microbiota on *Clostridium difficile* spore formation and germination. PLoS Pathog. 2015;11:e1005157. 26468647 10.1371/journal.ppat.1005157 PMC4607366

[bib150] Shida K, Makino K, Morishita A et al. *Lactobacillus casei* inhibits antigen-induced IgE secretion through regulation of cytokine production in murine splenocyte cultures. Int Arch Allergy Immunol. 1998;115:278–87. 9566350 10.1159/000069458

[bib149] Shi Y-C, Guo H, Chen J et al. Initial meconium microbiome in Chinese neonates delivered naturally or by cesarean section. Sci Rep. 2018;8:3255. 29459704 10.1038/s41598-018-21657-7 PMC5818670

[bib151] Simonyté Sjödin K, Hammarström ML, Rydén P et al. Temporal and long-term gut microbiota variation in allergic disease: a prospective study from infancy to school age. Allergy. 2019;74:176–85. 29786876 10.1111/all.13485

[bib152] Sjögren YM, Jenmalm MC, Böttcher MF et al. Altered early infant gut microbiota in children developing allergy up to 5 years of age. Clin Exp Allergy. 2009a;39:518–26. 19220322 10.1111/j.1365-2222.2008.03156.x

[bib153] Sjögren YM, Tomicic S, Lundberg A et al. Influence of early gut microbiota on the maturation of childhood mucosal and systemic immune responses: gut microbiota and immune responses. Clin Exp Allergy. 2009b;39:1842–51. 19735274 10.1111/j.1365-2222.2009.03326.x

[bib154] Starling AP, Sauder KA, Kaar JL et al. Maternal dietary patterns during pregnancy are associated with newborn body composition. J Nutr. 2017;147:1334–9. 28539412 10.3945/jn.117.248948 PMC5483965

[bib155] Stensballe LG, Simonsen J, Jensen SM et al. Use of antibiotics during pregnancy increases the risk of asthma in early childhood. J Pediatr. 2013;162:832–8.e3. 23140881 10.1016/j.jpeds.2012.09.049

[bib156] Stewart CJ, Ajami NJ, O'Brien JL et al. Temporal development of the gut microbiome in early childhood from the TEDDY study. Nature. 2018;562:583–8. 30356187 10.1038/s41586-018-0617-x PMC6415775

[bib157] Stewart CJ, Embleton ND, Clements E et al. Cesarean or vaginal birth does not impact the longitudinal development of the gut microbiome in a cohort of exclusively preterm infants. Front Microbiol. 2017;8:1008. 28634475 10.3389/fmicb.2017.01008 PMC5459931

[bib158] Strati F, Di Paola M, Stefanini I et al. Age and gender affect the composition of fungal population of the human gastrointestinal tract. Front Microbiol. 2016;7:1227. 27536299 10.3389/fmicb.2016.01227 PMC4971113

[bib159] Stuebe A The risks of not breastfeeding for mothers and infants. Rev Obstet Gynecol. 2009;2:222–31. 20111658 PMC2812877

[bib160] Talati A, Odgerel Z, Wickramaratne PJ et al. Brain derived neurotrophic factor moderates associations between maternal smoking during pregnancy and offspring behavioral disorders. Psychiatry Res. 2016;245:387–91. 27611068 10.1016/j.psychres.2016.08.061 PMC5067210

[bib161] Tamburini S, Shen N, Wu HC et al. The microbiome in early life: implications for health outcomes. Nat Med. 2016;22:713–22. 27387886 10.1038/nm.4142

[bib162] Tan H, Zhai Q, Chen W Investigations of *Bacteroides* spp. towards next-generation probiotics. Food Res Int. 2019;116:637–44. 30716990 10.1016/j.foodres.2018.08.088

[bib163] Tapiainen T, Koivusaari P, Brinkac L et al. Impact of intrapartum and postnatal antibiotics on the gut microbiome and emergence of antimicrobial resistance in infants. Sci Rep. 2019;9:10635. 31337807 10.1038/s41598-019-46964-5 PMC6650395

[bib164] Tetz G, Tetz V Introducing the sporobiota and sporobiome. Gut Pathog. 2017;9:38. 28680484 10.1186/s13099-017-0187-8 PMC5493122

[bib165] Turroni F, Milani C, Duranti S et al. Glycan utilization and cross-feeding activities by Bifidobacteria. Trends Microbiol. 2018;26:339–50. 29089173 10.1016/j.tim.2017.10.001

[bib166] Vallès Y, Artacho A, Pascual-García A et al. Microbial succession in the gut: directional trends of taxonomic and functional change in a birth cohort of Spanish infants. PLos Genet. 2014;10:e1004406. 24901968 10.1371/journal.pgen.1004406 PMC4046925

[bib167] van Nimwegen FA, Penders J, Stobberingh EE et al. Mode and place of delivery, gastrointestinal microbiota, and their influence on asthma and atopy. J Allergy Clin Immunol. 2011;128:948–55.e3. 21872915 10.1016/j.jaci.2011.07.027

[bib168] Vatanen T, Franzosa EA, Schwager R et al. The human gut microbiome in early-onset type 1 diabetes from the TEDDY study. Nature. 2018;562:589–94. 30356183 10.1038/s41586-018-0620-2 PMC6296767

[bib169] Vatanen T, Kostic AD, d'Hennezel E et al. Variation in microbiome LPS immunogenicity contributes to autoimmunity in humans. Cell. 2016;165:842–53. 27133167 10.1016/j.cell.2016.04.007 PMC4950857

[bib170] Vinturache AE, Gyamfi-Bannerman C, Hwang J et al. Maternal microbiome—a pathway to preterm birth. Semin Fetal Neonatal Med. 2016;21:94–9. 26936188 10.1016/j.siny.2016.02.004

[bib171] Wampach L, Heintz-Buschart A, Fritz JV et al. Birth mode is associated with earliest strain-conferred gut microbiome functions and immunostimulatory potential. Nat Commun. 2018;9:5091. 30504906 10.1038/s41467-018-07631-x PMC6269548

[bib172] Wang L-T, Lee F-L, Tai C-J et al. Comparison of *gyrB* gene sequences, 16S rRNA gene sequences and DNA–DNA hybridization in the *Bacillus subtilis* group. Int J Syst Evol Microbiol. 2007;57:1846–50. 17684269 10.1099/ijs.0.64685-0

[bib173] Wang M, Karlsson C, Olsson C et al. Reduced diversity in the early fecal microbiota of infants with atopic eczema. J Allergy Clin Immunol. 2008;121:129–34. 18028995 10.1016/j.jaci.2007.09.011

[bib174] Wang S, Ryan CA, Boyaval P et al. Maternal vertical transmission affecting early-life microbiota development. Trends Microbiol. 2020;28:28–45. 31492538 10.1016/j.tim.2019.07.010

[bib175] Wang X, Buhimschi CS, Temoin S et al. Comparative microbial analysis of paired amniotic fluid and cord blood from pregnancies complicated by preterm birth and early-onset neonatal sepsis. PLoS One. 2013;8:e56131. 23437088 10.1371/journal.pone.0056131 PMC3577789

[bib176] Ward TL, Knights D, Gale CA Infant fungal communities: current knowledge and research opportunities. BMC Med. 2017;15:30. 28190400 10.1186/s12916-017-0802-z PMC5304398

[bib177] Wen L, Ley RE, Volchkov PY et al. Innate immunity and intestinal microbiota in the development of Type 1 diabetes. Nature. 2008;455:1109–13. 18806780 10.1038/nature07336 PMC2574766

[bib178] WHO (World Health Organization) Appropriate technology for birth. Lancet. 1985;2:436–7. 2863457

[bib179] WHO (World Health Organization) Preterm Birth: Fact Sheet. https://www.who.int/en/news-room/fact-sheets/detail/preterm-birth (26 November 2019, date last accessed).

[bib180] Yassour M, Jason E, Hogstrom LJ et al. Strain-level analysis of mother-to-child bacterial transmission during the first few months of life. Cell Host Microbe. 2018;24:146–54.e4. 30001517 10.1016/j.chom.2018.06.007 PMC6091882

[bib181] Yassour M, Vatanen T, Siljander H et al. Natural history of the infant gut microbiome and impact of antibiotic treatment on bacterial strain diversity and stability. Sci Transl Med. 2016;8:343ra81. 10.1126/scitranslmed.aad0917 PMC5032909 27306663

[bib182] Yatsunenko T, Rey FE, Manary MJ et al. Human gut microbiome viewed across age and geography. Nature. 2012;486:222–7. 22699611 10.1038/nature11053 PMC3376388

[bib183] Zhang G, Feenstra B, Bacelis J et al. Genetic associations with gestational duration and spontaneous preterm birth. N Engl J Med. 2017;377:1156–67. 28877031 10.1056/NEJMoa1612665 PMC5561422

[bib184] Zhao D, Su H, Cheng J et al. Prenatal antibiotic use and risk of childhood wheeze/asthma: a meta-analysis. Pediatr Allergy Immunol. 2015;26:756–64. 26126682 10.1111/pai.12436

[bib185] Zijlmans MA, Korpela K, Riksen-Walraven JM et al. Maternal prenatal stress is associated with the infant intestinal microbiota. Psychoneuroendocrinology. 2015;53:233–45. 25638481 10.1016/j.psyneuen.2015.01.006

